# The ability in managing reactive oxygen species affects *Escherichia coli* persistence to ampicillin after nutrient shifts

**DOI:** 10.1128/msystems.01295-24

**Published:** 2024-10-29

**Authors:** Ruixue Zhang, Christopher Hartline, Fuzhong Zhang

**Affiliations:** 1 Department of Energy, Environmental & Chemical Engineering, Washington University in St. Louis, St. Louis, Missouri, USA; 2 Division of Biological and Biomedical Sciences, Washington University in St. Louis, St. Louis, Missouri, USA; 3 Institute of Materials Science and Engineering, Washington University in St. Louis, St. Louis, Missouri, USA; Northwestern University, Evanston, Illinois, USA

**Keywords:** nutrient shift, antibiotic persistence, reactive oxygen species, stress response

## Abstract

Bacterial persistence profoundly impacts biofilms, infections, and antibiotic effectiveness. Persister formation can be substantially promoted by nutrient shift, which commonly exists in natural environments. However, mechanisms that promote persister formation remain poorly understood. Here, we investigated the persistence frequency of *Escherichia coli* after switching from various carbon sources to fatty acid and observed drastically different survival rates. While more than 99.9% of cells died during a 24-hour ampicillin (AMP) treatment after the glycerol to oleic acid (GLY → OA + AMP) shift, a surprising 56% of cells survived the same antibiotic treatment after the glucose to oleic acid (GLU → OOA + AMP) shift. Using a combination of single-cell imaging and time-lapse microscopy, we discovered that the induction of high levels of reactive oxygen species (ROS) by AMP is the primary mechanism of cell killing after switching from gluconeogenic carbons to OA + AMP. Moreover, the timing of the ROS burst is highly correlated (*R*^2^ = 0.91) with the start of the rapid killing phase in the time-kill curves for all gluconeogenic carbons. However, ROS did not accumulate to lethal levels after the GLU → OA + AMP shift. We also found that the overexpression of the oxidative stress regulator and ROS detoxification enzymes strongly affects the amounts of ROS and the persistence frequency following the nutritional shift. These findings elucidate the different persister frequencies resulting from various nutrient shifts and underscore the pivotal role of ROS. Our study provides insights into bacterial persistence mechanisms, holding promise for targeted therapeutic interventions combating bacterial resistance effectively.

**IMPORTANCE:**

This research delves into the intriguing realm of bacterial persistence and its profound implications for biofilms, infections, and antibiotic efficacy. The study focuses on *Escherichia coli* and how the switch from different carbon sources to fatty acids influences the formation of persister-resilient bacterial cells resistant to antibiotics. The findings reveal a striking variation in survival rates, with a significant number of cells surviving ampicillin treatment after transitioning from glucose to oleic acid. The key revelation is the role of reactive oxygen species (ROS) in cell killing, particularly after switching from gluconeogenic carbons. The timing of ROS bursts aligns with the rapid killing phase, highlighting the critical impact of oxidative stress regulation on persistence frequency. This research provides valuable insights into bacterial persistence mechanisms, offering potential avenues for targeted therapeutic interventions to combat bacterial resistance effectively.

## INTRODUCTION

Bacterial persistence is a critical factor contributing to antibiotic treatment failure, biofilm formation, bacterial resistance, and the recurrence of infections, significantly impacting the effectiveness of antibiotic therapies (1, 2). Bacterial persistence involves phenotypic heterogeneity within bacterial populations, delineating a subset of cells termed “persisters” that exhibit antibiotic tolerance, while the remaining majority remains susceptible to antibiotics (3). Persisters are non-heritable phenotypic variants characterized by slow growth or non-growing dormancy (4–6). While stochastically formed persisters are rare, a substantial number of persisters can form in the stationary phase (6). Moreover, various environmental stressors, including antibiotic exposure, osmotic pressure, extreme temperatures, genetic mutations, and nutrient deprivation, have been identified as triggers for persister formation (7–9). The levels of triggered persisters exhibit substantial variability contingent upon culture conditions, bacterial species, and the strength of the stressor. Due to the presence of two distinct subpopulations, applying antibiotics to a persister-enriched population typically produces a biphasic killing curve (6): an initial rapid killing phase targeting susceptible cells, succeeded by a slower killing phase affecting the persistent subpopulation. This biphasic killing curve stands as a hallmark of bacterial persistence, with the transition between the two killing phases indicating the size of the persistent subpopulation (10).

Persister cells survive antibiotic treatment through antibiotic target inactivity and dormancy caused by energy loss, halted DNA replication, and blocked translation (11). However, the complexities of persister cells go beyond just inactivity and dormancy. Some persister cells use intrinsic mechanisms to mitigate antibiotic-inflicted damage or lower the concentration of antibiotics within the cell (12). Various mechanisms for persister formation have been suggested and investigated, such as the stringent response via (p)ppGpp and the SOS response, although there still remain nuances and debates on the roles and specifics of each system in persister formation (13, 14). Furthermore, heterogeneity in secondary messengers, such as cAMP (15), metabolic activities (16, 17), and energy molecules ATP and GTP also play vital roles in persister formation (18).

Bacterial populations constantly confront metabolic stress owing to their existence in a dynamically changing environment. However, the comprehension of stress-triggered persister cell formation still needs to be completed. The persistence that emerged due to nutrient shifts has garnered considerable attention, given the frequent occurrence of such shifts in natural settings. Notably, diauxic nutrient shifts have been observed to elevate the frequency of persisters (19–21). In the case of a complete nutrient shift from glucose to fumarate, an *Escherichia coli* subpopulation was shown to be mostly tolerant to ampicillin (AMP) (17). However, when switching *E. coli* from a gluconeogenic carbon, including pyruvate, glycerol, succinate (SUC), malate (MAL), or acetate (ACE), to fatty acid (FA), a non-typical tri-phasic antibiotic-killing kinetics were recently observed (22). In these cases, cells exhibit a transient period of high antibiotic tolerance without cell death in the presence of ampicillin, followed by rapid killing of sensitive cells and a slow killing of persisters formed from the previous culture. The duration of the transient tolerance varies with different gluconeogenic carbons and correlates with the time needed to express the FA degradation enzymes during each nutrient shift.

**Fig 1:**
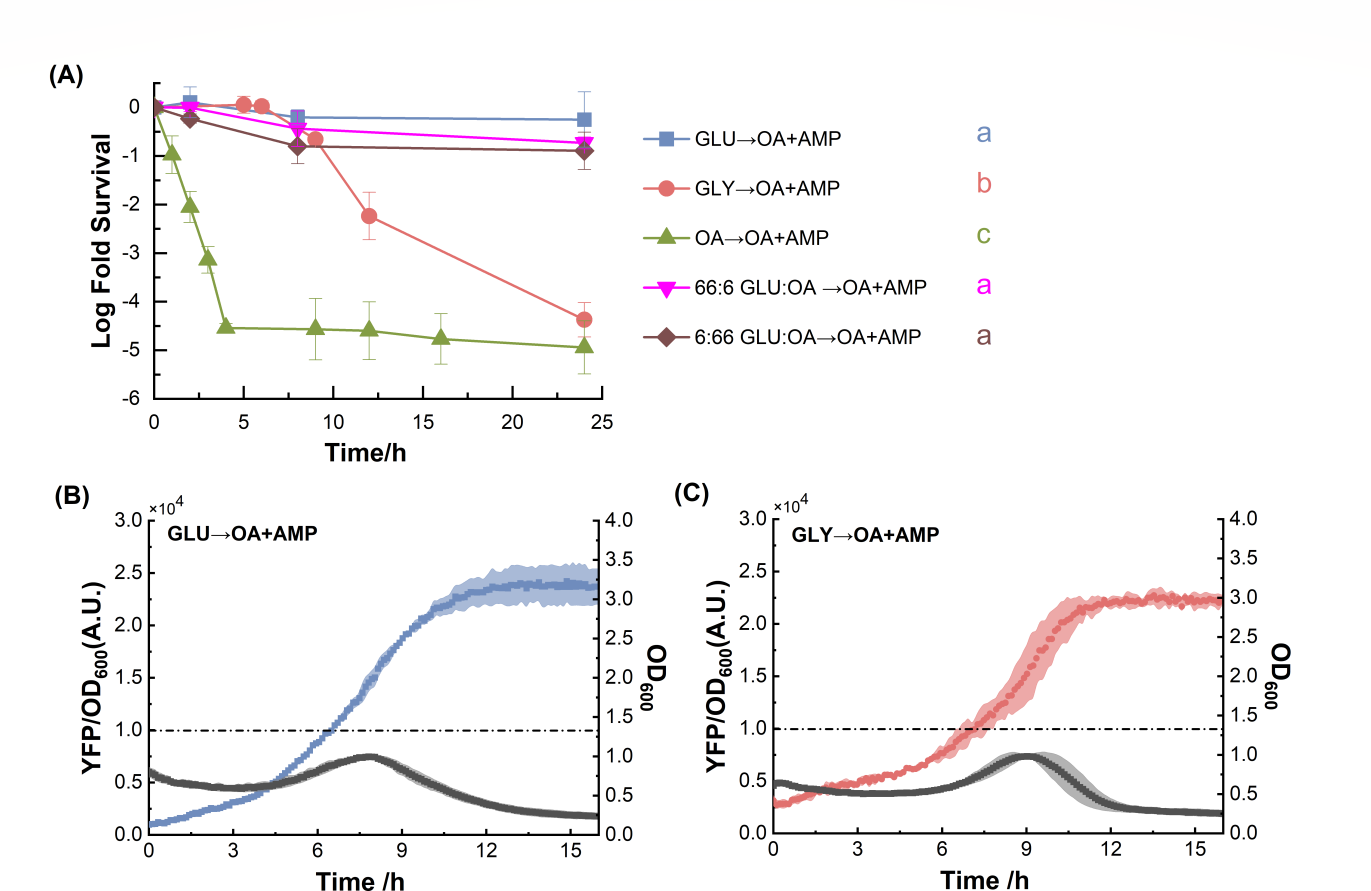
Nutrient shifts from glucose or glycerol to OA with ampicillin (OA + AMP) triggered drastically different persister levels. (**A**) Time-kill curves of *E. coli* after nutrient shifts from glucose or glycerol to OA + AMP. Numbers in the insert represent the carbon molar ratio of glucose over OA with a total carbon fixed at 72 mM. Statistical results were calculated using a pairwise comparison method with Tukey’s test of the area (between 0 and 24 hours) under the curve, and the results are presented with colored lowercase letters next to each curve. (**B and C**) Time course of yellow fluorescent protein (YFP)/OD_600_ from FadD-YFP fusion (colored line, left axis) mutant strain and OD_600_ (gray dotted line, right axis) after GLU → OA + AMP (**B**) and GLY → OA + AMP (**C**) shifts. The black dash lines in (**B**) and (**C**) indicate the YFP/OD_600_ threshold, after which FadD was accumulated to a level which is correlated with resuming exponential growth after a lag phase (see Materials and Methods).

The varying quantities of persisters identified in preceding studies prompted our investigation into the molecular mechanisms of cell survival amid nutrient transitions. In this study, we observed starkly contrasting patterns of cell-killing kinetics upon switching *E. coli* cells from glucose (GLU) or glycerol (GLY) to fatty acids (e.g., oleic acid, OA) supplemented with ampicillin. Notably, while the glycerol to OA (GLY → OA + AMP) shift triggered the transient tolerance followed by over 99.9% of cells killed within 24 hours incubation in ampicillin, the shift from glucose to OA (GLU → OA + AMP) resulted in 56% of cells being persistent even after a 24-hour exposure to ampicillin (Fig. 1A). Using a combination of single-cell imaging and time-lapse microscopy, we uncovered that the induction of reactive oxygen species (ROS) by ampicillin serves as the primary mechanism for cell death after nutrient shift from gluconeogenic carbons to OA. Specifically, we found that the number of cells with elevated intracellular ROS concentrations surged after the GLY → OA + AMP shift but not after the GLU → OA + AMP shift. Moreover, our investigations demonstrated that the expression level of the oxidative stress regulator (encoded by *oxyR*) and ROS detoxification enzymes (encoded by *katG*, *sodA,* and *ahpCF*, respectively) mediates cellular ROS levels and strongly affects persister formation after nutrient shift. These findings explained the disparate levels of persisters arising from various nutrient shifts in *E. coli* and underscored the pivotal role of ROS in mediating persister formation induced by nutrient shifts.

## RESULTS

### Nutrient shifts from glucose or glycerol to OA triggered drastically different persister levels

Glucose, a prevalent end product of dietary carbohydrates, stands as one of the most abundant (23) and preferred (24) carbon sources for *E. coli* within its natural habitats. Additionally, fatty acids and glycerol, derived from the breakdown of dietary lipids, also commonly exist in the natural environments of *E. coli* (25). Transitions between these diverse carbon sources occur frequently, prompting *E. coli* to evolve metabolic adaptation mechanisms for such shifts (26). Using the wild-type NCM3722 *E. coli* strain, we first grew the cells in minimal glucose medium to exponential growth phase (Fig. S1; Table S1) and switched to minimal OA medium supplemented with ampicillin (GLU → OA + AMP). Cells were then taken from the OA + AMP culture at various time points and transferred to Luria-Bertani (LB) agar plates for resuscitation after washing out ampicillin. We found that most cells became persisters in the OA + AMP culture, and they resumed growth on LB agar plates upon ampicillin removal (Fig. 1A). Even after a 48-hour incubation in OA + AMP, a surprising 20% of cells survived. In contrast, when *E. coli* grown in minimal glycerol medium was switched to OA medium supplemented with ampicillin (GLY → OA + AMP), a substantial proportion of cells was killed rapidly after a transient tolerant period, leaving only a tiny fraction (~10^−4^) of persister cells after the 24-hour ampicillin exposure (Fig. 1A), aligning with previous findings (22). Meanwhile, adding the same amount of AMP to *E. coli* cultures exponentially growing in either glucose or OA led to immediate cell killing, eliminating 99.9% of the population by 24 hours (Fig. 1A). Furthermore, without ampicillin, cells grow normally in minimal OA medium after switching from glucose or glycerol (Fig. S2).

To maintain consistency with previous studies, a washing step using ice-cold nutrient-free media was employed during nutrient shifts (22, 27, 28). This procedure ensures the effective elimination of any residual amount of pre-shift nutrients, which might otherwise be transferred to the new nutrient. To investigate whether high persistence can be obtained from a more gradual GLU → OA + AMP shift, we supplemented different amounts of OA to the pre-shift M9 glucose medium and evaluated the persistence levels. Although adding OA to the pre-shift culture reduced the persister levels to 10% for 66:6 GLU:OA → OA + AMP and 6% for 6:66 GLU:OA → OA + AMP after 48-hour ampicillin treatment, it still produced 1,000-fold more persisters compared to that from the GLY → OA + AMP shift (Fig. 1A). These results underscore the robust persister formation mechanism after GLU → OA + AMP, which is not strongly affected by switching methods.

*E. coli* employs carbon catabolite repression to prevent the simultaneous metabolism of glucose alongside other carbon sources such as FA (29). Previous studies found that the transient tolerance period after a nutrient shift from gluconeogenic carbon to OA was caused by slow accumulation of the FA degradation enzymes (β-oxidation) (22). Together, these facts motivated us to investigate whether the high persistence after 48 hours of GLU → OA + AMP is caused by the lack of expression on β-oxidation enzymes and poor ability to metabolize OA, which finally led to an unexpectedly long transient tolerance period and a high persistent subpopulation. To address this, we monitored the expression of the β-oxidation pathway by fusing a yellow fluorescent protein (YFP) in the same operon downstream of *fadD*, which encodes the sole copy of acyl-CoA synthetase (FadD) in *E. coli*. FadD catalyzes the rate-limiting step in β-oxidation and serves as an indicator of FA metabolism (26, 30). Upon nutrient shifts from both glucose and glycerol to OA, the kinetics of FadD expression exhibited striking similarities (Fig. 1B and C). FadD was not expressed to sufficient levels until 6–7 hours after the nutrient shift, determined according to the previous method (22), and shown as the short dash dotted line in Fig. 1B and C. Prior to this time frame, cells demonstrated insufficient FA metabolic activity to support growth, thereby evading cell death due to growth arrest. After 5–6 hours, cells from both shifts have accumulated sufficient β-oxidation pathway enzymes to facilitate FA metabolism. This leads to rapid cell killing by ampicillin for cells from the GLY → OA + AMP shift, consistent with our previous observation (22). However, these analogous FadD expression patterns fail to elucidate the observed discrepancy in persistence frequency, as evidenced in time-kill experiments (Fig. 1A).

### ROS participated in cell killing after nutrient shift

**Fig 2:**
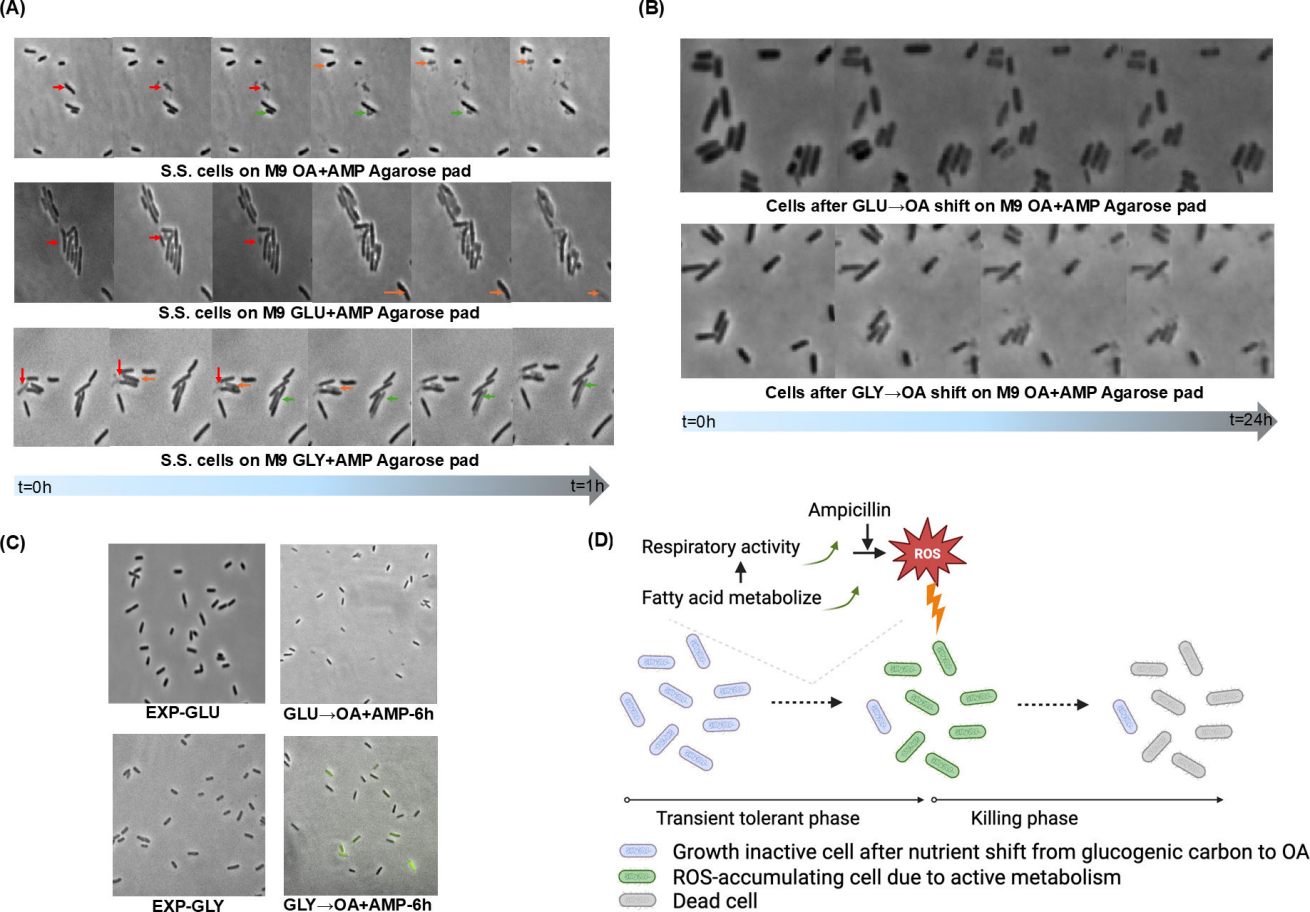
ROS is involved in cell killing after nutrient shifts to OA + AMP. (**A**) Time-lapse images of steady state (S.S.) *E. coli* cells treated with 100 µg/mL ampicillin in different media. Top, M9 OA; medium, M9 glucose; bottom, M9 glycerol. The colored arrows point to cells lysed during ampicillin treatment. Each color represents one cell. (**B**) Time-lapse images of cells after the nutrient shift from M9 glucose (top) or M9 glycerol (bottom) to M9 OA + AMP on agarose pad. (**C**) Images of *E. coli* cells from different stages stained with carboxy-H2DCFDA. Green fluorescence represents relative intracellular ROS intensity. Top left, log-phase cells grown on M9 glucose; bottom left, log-phase cells grown on M9 glycerol; top right, cells at 6 hours after GLU → OA + AMP; bottom right, cells at 6 hours after GLU → OA + AMP. EXP, exponentially growing cells. (**D**) Experimental procedure and proposed mechanism.

Subsequently, employing time-lapse microscopy, we examined the manner in which ampicillin induced cell death subsequent to the GLU → OA + AMP shift. The introduction of ampicillin into log-phase *E. coli* glucose, glycerol, or OA culture induced cell lysis, evidenced by conspicuous disruptions in cell outlines captured in the phase-contrast images (Fig. 2A). Intriguingly, no apparent cell lysis was observed post-nutrient shifts from glucose or glycerol to OA (Fig. 2B). Even after a 24-hour incubation in an ampicillin-infused OA medium following the nutrient shift, cells originating from the GLY → OA + AMP shift remained structurally intact despite the effective elimination of cell viability for 99.99% of cells. Ampicillin’s principal mechanism of inducing cell death is through the inhibition of cell wall biosynthesis (31), resulting in compromised cell walls and cell lysis. While ampicillin efficiently eradicates log-phase *E. coli* cells via this primary mechanism, the absence of observable cell lysis after the nutrient shift to OA suggests the involvement of an alternative mechanism in inducing cell death.

Antibiotics possess an additional mode of bactericidal action involving reactive oxygen species (32–34). Bacteria naturally produce limited quantities of ROS, including hydrogen peroxide, hydroxyl radical, singlet oxygen, and superoxide anion, during oxidative respiration (35). Exposure to antibiotics or environmental stressors can significantly escalate cellular ROS levels (36), with ampicillin specifically shown to elevate these levels (33). Elevated ROS levels lead to detrimental effects on proteins, lipids, and nucleic acids, setting off a cascade that amplifies ROS production and eventually culminating in cell death without lysis (37). We hypothesized that post a GLY → OA + AMP shift, cells were killed due to amplified cellular ROS concentrations induced by ampicillin. To validate this hypothesis, we stained the cells following the nutrient shift using carboxy-H2DCFDA, a fluorescent dye commonly employed for assessing cellular ROS levels. Within the initial 5 hours after the GLY → OA + AMP shift, cells exhibited a low level of fluorescence during the transient tolerant period. However, at the 6-hour mark after the nutrient shift, a significant fraction of cells displayed intense fluorescence, indicative of ROS accumulation (Fig. 2C). The timing of the ROS burst after the GLY → OA + AMP switch matches with time-kill kinetics and the expression pattern of FadD, suggesting an alternative killing mechanism (Fig. 2D). Immediately after the GLY → OA + AMP switch (i.e., the first 6 hours), cells slowly adapt to the new OA carbon source by gradually expressing β-oxidation enzymes, and its metabolism remains inactive. During this period, cells do not grow, their ROS levels are low due to inactive oxidative respiration, and they are transiently tolerant to ampicillin. However, by the 6-hour mark of the nutrient shift, the levels of FadD surpassed a threshold necessary for adequate flux through OA metabolism. Concurrently, the presence of ampicillin triggered increased ROS formation through active oxidative respiration. Cells accumulating lethal ROS levels were consequently killed without undergoing cell lysis. By the 9-hour mark post-shift, a majority of cells had been killed by this mechanism and did not manifest intense green fluorescence indicative of ROS accumulation.

**Fig 3:**
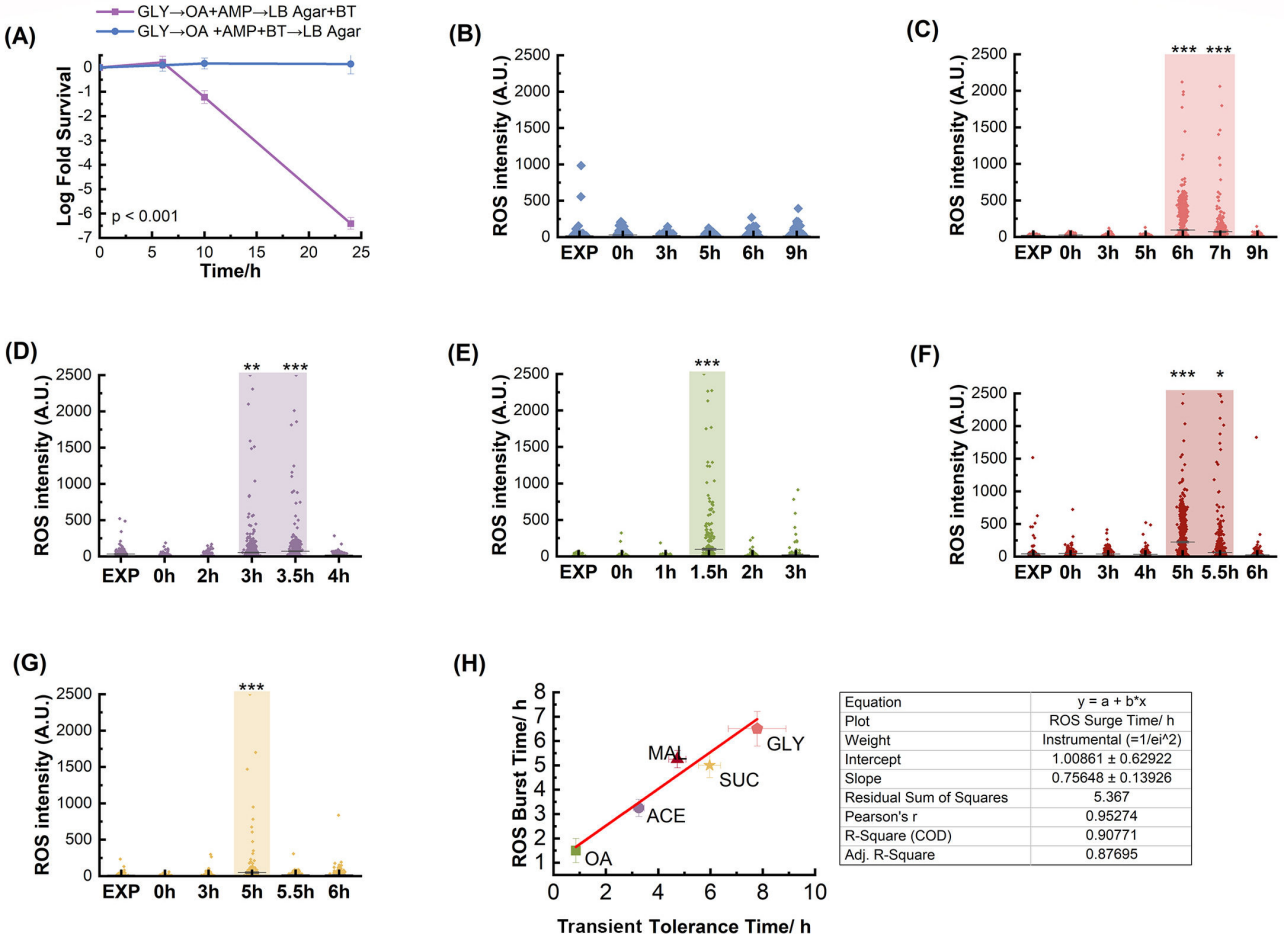
ROS accumulation after nutrient shift from glycerol to OA + AMP. (**A**) Time-kill curves of *E. coli* after nutrient shifts from glycerol to OA + AMP. ROS scavenger, BT, was added either during the ampicillin treatment (blue) period or during the resuscitation period in LB agar plate (purple). Statistical results were calculated using a pairwise comparison method with Tukey’s test. (B–G) Relative ROS intensity in single cells (*n* >300 from biological triplicates) at different time points after the nutrient shift from glucose (**B**), glycerol (**C**), acetate (**D**), OA (**E**), malate (**F**), and succinate (**G**) to OA + AMP. Each dot represents a single cell. *P* values were calculated using a pairwise comparison method with Holm-Sidak’s test, and the results were presented as asterisks for (B–G). (**H**) Correlation between the onset of transient tolerance period and the ROS burst time. Pearson’s correlation coefficient (*R*) was calculated, and curve fitting for (**H**) was analyzed with the result shown on the right.

To further confirm that cells following the GLY → OA + AMP transition were killed by the secondary ROS-mediated damage, we introduced the ROS scavenger, BT (comprising 0.28 mM bipyridyl plus 90 mM thiourea), into the time-kill kinetics assay. By impeding ROS generation during ampicillin exposure, nearly 100% of cells managed to survive even after a 24-hour incubation in ampicillin subsequent to the GLY → OA + AMP shift (Fig. 3A). This outcome unequivocally dismisses the possibility of post-stress cell death (38) and affirms the pivotal role of ROS in antibiotic-induced cell death following the GLY → OA + AMP shift.

### ROS bursts match the onset of the rapid killing phase after gluconeogenic to OA shift

We noted that cells after the GLU → OA + AMP shift showed a diminished ROS level even after the FadD threshold required for cell growth in the M9 OA medium (Fig. 3B). This restrained ROS level, indicative of the absence of an ROS-mediated secondary cell-killing mechanism, is consistent with the elevated cell survival rate following the GLU → OA shift. Simultaneously, we noticed an ROS surge at the end of transient tolerant period for GLY → OA + AMP shift (Fig. 3C).

We further postulated that ROS-induced cell death following nutrient shifts from various gluconeogenic carbons to OA occurs through a comparable mechanism to that observed in the GLY → OA + AMP shift (Fig. 3C). To investigate this, we quantified ROS intensity at distinct time intervals after nutrient shifts from MAL, SUC, ACE, and OA to OA + AMP, comparing them with ROS levels in log-phase cells grown on each respective carbon source (Fig. 3D through G). Analogous to the GLY → OA + AMP shift, ROS levels remained consistent with pre-shift levels immediately after the nutrient shift across all carbon sources due to the requisite duration for β-oxidation enzymes and ROS accumulation. Subsequently, bursts of ROS were detected in certain cells at later time points, with variations in the timing of these bursts corresponding to different carbon sources. The duration until ROS burst following each gluconeogenic carbon to OA shift broadly aligned with the commencement of their rapid cell-killing phases delineated by time-kill curves (Fig. 3H). For instance, the ROS surge occurred between 3 and 3.5 hours after the ACE → OA + AMP shift (Fig. 3D), correlating with its early onset of the rapid killing phase (3.3 ± 0.2 hours). Notably, in the case of OA → OA + AMP transfer without nutrient shift, significant ROS accumulation transpired swiftly (Fig. 3E), consistent with rapid killing soon after nutrient shift. In summary, our findings suggest that the accumulation of ROS to lethal concentrations stands as the underlying mechanism governing cell death after nutrient shifts from gluconeogenic carbons to OA.

### Persister control by targeting oxidative stress regulator and ROS detoxification enzymes

We next targeted pinpointing the mechanism facilitating cells in controlling ROS levels at non-lethal thresholds subsequent to the GLU → OA + AMP shift. Bacteria possess a repertoire of enzymes and pathways aimed at combating oxidative stress induced by ROS (39). The oxidative stress regulator, OxyR (encoded by *oxyR*), stands as a pivotal oxidative stress-specific transcription factor governing numerous antioxidant genes. Previous investigations have unveiled the indispensable roles of OxyR in mitigating ROS, safeguarding DNA, and repairing damaged proteins (39–41). OxyR upregulates multiple ROS detoxification enzymes in *E. coli*, including a catalase (encoded by *katG*), an alkyl hydroperoxide reductase (encoded by *ahpCF*), and a superoxide dismutase (encoded *sodA*), all of which participate in ROS detoxification reactions (39).

**Fig 4:**
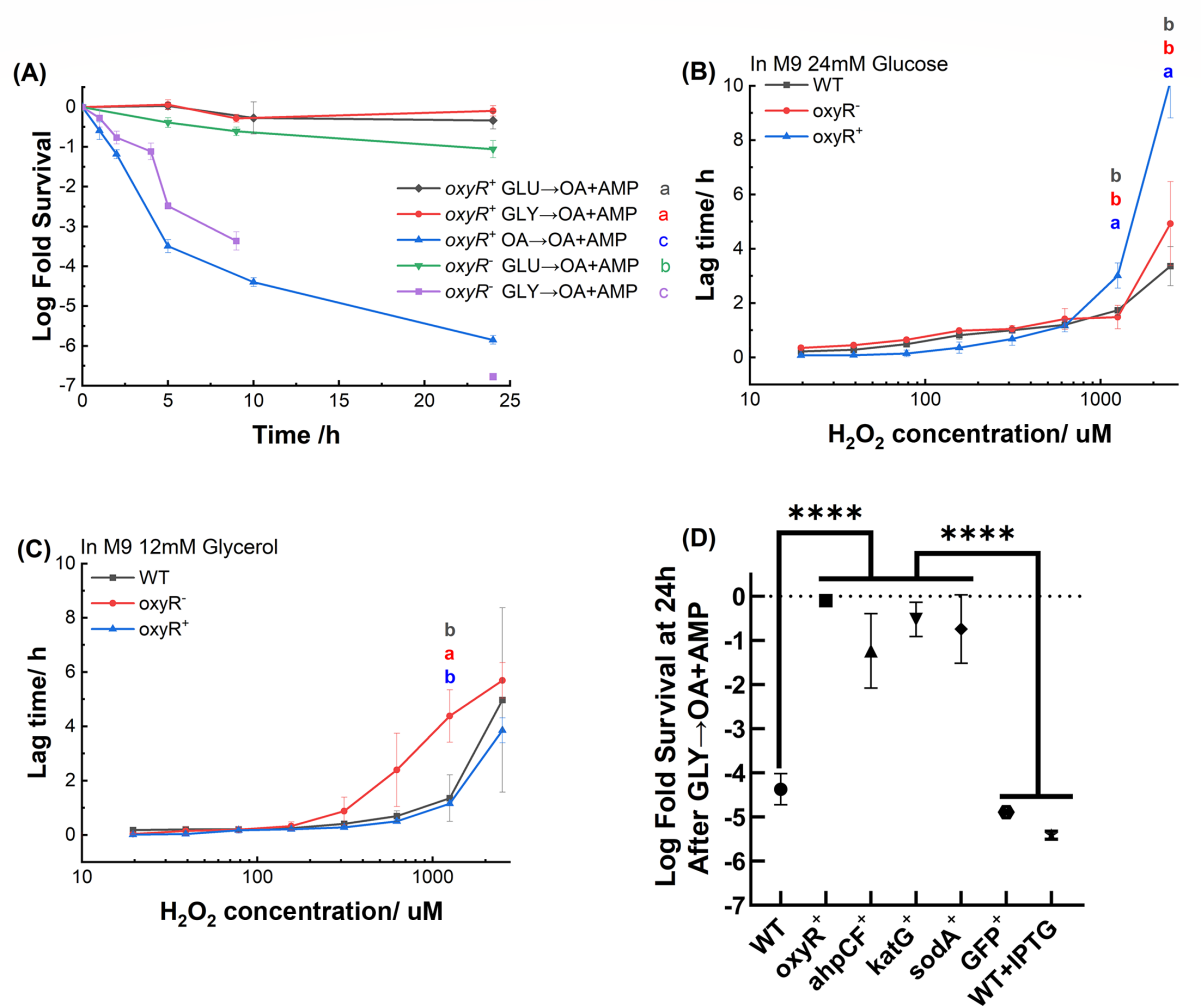
The ability to control oxidative stress affects the number of persisters to ampicillin after nutrient shifts. (**A**) Time-kill curves of the *oxyR* overexpression mutant (*oxyR*^+^) and *oxyR*-deficient mutant (*oxyR*^−^) after nutrient switch experiments. Statistical results were calculated using a pairwise comparison method with Tukey’s test of the area under the curve, and the results are presented with colored lowercase letters. Cells were treated with various concentrations of H_2_O_2_ followed by cultivation in M9 glucose (**B**) or M9 glycerol (**C**). The gray line represents WT *E. coli*, the red line represents *oxyR^−^* mutant, and the blue line represents *oxyR^+^* mutant. At each H_2_O_2_ concentration, statistical results were calculated using a pairwise comparison method with Tukey’s test, with the results displayed as lowercase letters next to each curve. (**D**) Comparison of log fold survival at 24 hours after the GLY → OA + AMP in strains overexpressing oxidative stress regulator (*oxyR*^+^) or detoxification enzymes (*ahpCF*^+^, *katG*^+^, and *sodA*^+^). The persister levels of the wild-type *E. coli* strain (WT), the GFP overexpression strain (*gfp*+), and the WT strain treated with IPTG (WT + IPTG) were selected as negative controls to rule out any potential effects from the plasmid vector, protein overexpression, or the inducer. Statistical results were calculated using a pairwise comparison method with Tukey’s test of survival rate at 24 hours.

To understand how OxyR modulates ROS levels during nutrient shifts, we created an *oxyR* knockout strain (*oxyR^−^*). Reduced cell survival rates were observed for the *oxyR^−^* mutant after both the GLU→OA + AMP and GLY→OA + AMP shifts, albeit to different degrees (Fig. 4A). In the case of GLY → OA + AMP shift, immediate cell death was observed without a transient tolerant period (Fig. 4A). At the 24-hour mark post the GLY → OA + AMP shift, the surviving cell number after ampicillin treatment was too meager for accurate colony counting (below the detection limit). Furthermore, the *oxyR*^−^ strain demonstrated reduced efficacy in managing ROS, evident from an extended lag phase following treatment with sub-lethal doses of H_2_O_2_ (Fig. 4B and C, red line with round marker). The extended lag phase is more apparent when cells are growing in glycerol (Fig. 4C) than in glucose (Fig. 4B), implying that *E. coli* has a more robust ROS management ability when growing in glucose compared to that in glycerol. Consequently, the deletion of *oxyR* led to a moderate reduction in cell survival after the GLU → OA + AMP shift, with approximately 10% of *oxyR*^−^ cells surviving a 24-hour ampicillin treatment. The low cellular ROS intensity (Fig. S3A and B) detected for *oxyR^−^* mutant is due to the majority of cells died at earlier time. The collective evidence strongly indicates that increased sensitivity of the *oxyR^−^* mutant to ROS leads to reduced cell survival from nutrient shifts.

Furthermore, we created an o*xyR* overexpression strain (*oxyR*^+^). While the time-kill kinetics following the GLU → OA + AMP shift displayed little difference between *oxyR*^+^ and the wild-type strains, a notable elevation in persistence emerged after the GLY → OA + AMP shift (Fig. 4A). Consistent with this enhanced persistence, a high level of ROS was not observed in *oxyR*^+^ cells after GLY → OA + AMP shift (Fig. S3C and D).

We further verified that the ROS-assisted killing can be mediated by overexpressing enzymes participating in ROS detoxification reactions (Fig. 4D). We found that overexpressing of either one of the ROS detoxification enzymes (encoded by *katG*, *ahpCF,* or *sodA*) leads to significantly elevated persister levels compared to the WT. This is consistent with previous observations that all three ROS detoxification enzymes are upregulated by OxyR (39). These outcomes imply that expressing OxyR or any one of the ROS detoxification enzymes can effectively enhance ROS management capability and maintain intracellular ROS levels at a minimum following the GLY → OA + AMP shift, thereby enabling cell survival from ROS-induced secondary damage. In essence, our findings underscore *E. coli*’s disparate abilities in ROS regulation when growing in glucose vs glycerol, resulting in distinct ROS management capacities that impact cell persistence post-nutrient shifts.

## DISCUSSION

Bacteria frequently encounter environmental fluctuations, disrupting the homeostasis of bacterial metabolism and impacting antibiotic treatment outcomes. Prior research delineated varying persister levels resulting from nutrient shifts. This study investigated into the mechanisms underlying diverse persistence phenotypes when switching from glucose or gluconeogenic carbon to FA. Our findings present compelling evidence linking variations in cellular ROS levels post-nutrient shifts to divergent outcomes in antibiotic efficacy. ROS has been recognized as a double-edged sword for bacterial killing (42). While lethal concentrations of ROS damage DNA, lipids, and proteins, leading to cell death (42), sub-lethal ROS was found to trigger persistence formation by reducing respiration, increasing the expression of the multidrug-resistant pump, or other mechanisms (43, 44). Antibiotics can kill bacteria through either a primary mechanism by directly damaging active cellular processes or a secondary mechanism by inducing ROS generation (45). Our data showed that the reduced metabolism at the lag phase immediately after nutrient shift (i.e., the transient tolerance period) prevents ampicillin from killing the bacteria via its primary mechanism. Cells are killed by ROS stimulated as a consequence of both increased respiration and ampicillin treatment at the end of the transient tolerant phase. Pre-shift nutrients determine the metabolism, which shapes the post-shift ROS accumulation. While cells from the gluconeogenic carbon to OA shift induced lethal levels of ROS, leading to cell death, cells from the GLU → OA + AMP shift managed to control their ROS to a non-lethal level, thus surviving from ampicillin exposure. Moreover, the transient tolerance period is controlled by the duration required for ROS to accumulate to lethal levels. Our findings not only explain persister level disparities arising from nutrient shifts but also underscore the intricate interplay of ROS in bacterial persistence and antibiotic responses. The metabolic differences between glucose and glycerol that lead to different ROS levels would require further studies at the system levels (17, 46).

Comprehending the key determinants governing persister formation in bacteria holds paramount importance in designing efficacious antibiotics to combat recurring and stubborn bacterial infections. This study illustrates the pivotal role of OxyR as well as ROS detoxification enzymes in modulating cellular ROS levels. The *oxyR^−^ E. coli* mutant displays increased susceptibility to ampicillin post-nutrient shifts. After GLY → OA + AMP shift, *oxyR* deletion eradicates the transient tolerance phase, rendering *E. coli* vulnerable to immediate post-shift elimination. Similarly, after the GLU → OA + AMP shift, *oxyR* deletion reduces the persister population to 10% at the 24-hour mark. Hence, employing an OxyR inhibitor in conjunction with antibiotics may serve as a potential strategy to bolster their efficacy. Moreover, the observation that a complete GLU → OA + AMP shift triggers persistence formation could offer valuable dietary insights into clinical antimicrobial interventions. Avoiding diets that induce GLU → OA + AMP shifts might reduce the emergence of antibiotic-tolerant bacteria. Lastly, our observations bear relevance in bioengineering contexts. When using engineered bacteria to produce valuable metabolite products (47–49), nutrient shifts during fermentation could provoke ROS accumulation, triggering adaptive cellular responses such as altering TCA cycle activity or enhancing mutation rates, thereby influencing bioproduction outcomes.

## MATERIALS AND METHODS

### Materials

Phusion DNA polymerase, restriction enzymes, and T4 ligase used in plasmid construction were purchased from Thermo Fisher Scientific (Waltham, MA, United States). DNA-spin Plasmid DNA Purification Kit and MEGA quick-spin Total DNA Purification Kit were purchased from iNtRON Biotechnology (Burlington, MA, USA). Antibiotics and inducers were purchased from Gold Biotechnology, Inc. (St. Louis, MO, USA). Other chemicals were purchased from Sigma-Aldrich (St. Louis, MO, USA).

### Strains, plasmids, and construction

The *E. coli* K-12 strain NCM3722, obtained from the Coli Genetic Stock Center (Yale, USA.), was used in this study. All strains and plasmids are listed in Table S2 and Table S3, respectively. *E. coli* MDS42pdu was used for cloning. DNA Golden Gate Assembly and BglBrick (50) were used for plasmid construction. Primers were designed by Benchling and synthesized by Integrated DNA Technologies (Coralville, IA, USA). Plasmids were transformed into electrocompetent cells by electroporation and selected on LB agar plates with corresponding antibiotics (ampicillin, 100 mg/L; kanamycin, 50 mg/L; streptomycin 100 mg/L, chloramphenicol, 25 µg/mL). Genome editing was conducted by following the pTarget-pCas homologous recombination protocol as described previously (51, 52). Colony PCR was used to select the desired mutants, whose sequences were further confirmed by Sanger sequencing. Protein overexpression plasmids used in this study were constructed by inserting the DNA sequence into an IPTG-inducible vector containing a low-copy number (SC101) origin of replication, chloramphenicol resistance, and LlacO-1 promoter.

### Culturing conditions

All strains were grown from single colonies and cultivated overnight in a Luria-Bertani medium before the experiments. Cells were then diluted into an M9 minimal medium supplemented with different carbon sources, including glycerol (24 mM), glucose (12 mM), oleic acid (4 mM), acetate (36 mM), succinate (18 mM), or malate (18 mM). Cells were cultivated at 37°C with 250 rpm of shaking until reaching steady state (at least six doublings of exponential phase growth). Steady-state cells were then collected for further assays. All cultures were supplemented with appropriate antibiotics (ampicillin, 100 mg/L; kanamycin, 50 mg/L; streptomycin, 100 mg/L; chloramphenicol, 25 µg/mL).

### Nutrient shift persistence assay

Nutrient shift protocols were developed following previous methods (22, 27). In brief, cells growing at 37°C in M9 media containing a carbon source were repeatedly diluted to a low cell density using the same media to maintain cell growth at the exponential growth phase. Cells were then collected and centrifuged in a pre-chilled centrifuge at 4,500 rcf for 10 minutes. Cells were then washed with ice-cold M90 medium (without carbon) three times. After washing, cells were resuspended into M9 oleic acid (4 mM) media containing 100 mg/L of ampicillin with an initial OD between 0.1 and 0.2. At various points after the nutrient shift to M9 OA supplemented with ampicillin media, 1 mL of culture was collected and centrifuged at 4,500 rcf, 4°C for 5 minutes. Cells were then washed with ice-cold 1× phosphate-buffered saline (PBS, pH 7.4) four times to remove ampicillin. Finally, cells were resuspended into PBS and subjected to a serial dilution. After each dilution, 5 µL of culture was plated onto an LB agar plate and incubated for 12 hours at 37°C. Colonies were counted to determine the colony-forming units per milliliter of culture. For strains with a protein overexpression plasmid, 1 mM IPTG was added to the pre-nutrient shift culture (M9 glycerol) when the cell OD_600_ reached 0.1 to overexpress the protein of interest. The cells were continuously induced with 1 mM IPTG for the remainder of the persister-killing assay.

### FadD-YFP expression kinetic assays

Gene expression kinetics were measured from a fluorescent plate reader following previous methods (53, 54). Specifically, log-phase cells cultivated in various pre-shift carbon sources were washed four times with M9-0 medium (without carbon source). Cells were then suspended in M9 OA media with or without ampicillin to an initial OD_600_ of 0.08 and transferred to a 96-Well Imaging Microplate (Corning, NY, USA) with a final volume of 150 µL per well. Cells were incubated inside an Infinite F200PRO plate reader (TECAN, Männedorf, Switzerland) at 37°C with consistent shaking. OD_600_ and fluorescence measurements (excitation: 514 nm, emission: 552 nm) were recorded every 6 minutes. Three wells containing M9-0 were used as negative controls. Final OD and YFP values were calculated by subtracting the average OD and YFP values from negative controls, respectively. Relative intracellular FadD concentration was calculated as YFP/OD_600_. The FadD threshold was previously determined by taking the average YFP/OD_600_ level at the end of the lag phase in the absence of antibiotic across all nutrient shift conditions (22).

### ROS measurements

To avoid introducing additional factors that may affect ROS levels during a nutrient shift persister experiment, the centrifugation condition and washing buffer used in this step are consistent with our nutrient shift persister assay. In brief, 1 mL of cell culture was collected by centrifugation and washed twice with PBS. The cells were then resuspended in 1 mL PBS containing 20 µM carboxy-H2DCFDA and incubated at 37°C in the dark for 20 minutes, followed by dye removal via centrifugation and resuspension in PBS. The labeled cells were then spread on a glass slide for imaging under a microscope. Fluorescence microscopy was performed using a Nikon Eclipse Ti microscope (Nikon Instruments Inc., USA) equipped with an EMCCD camera (Photometrics Inc., Huntington Beach, CA, USA) and a 100×, NA 1.40, oil-immersion phase-contrast objective lens. An X-Cite 120 LED was used as the light source. The FITC (Nikon Instruments Inc., USA) filter cube was used for spectral separation. The power of the LED light was carefully controlled so that no significant photobleaching was detected. Images were collected by an automated scanning function of the microscope with a built-in perfect focus system and analyzed using the Nikon NIS-elements software package. More than 300 single cells per sample were collected and analyzed.

### Time-lapse imaging

Cell cultures were collected and centrifuged at 6,500 g, 4°C for 5 minutes. The supernatant was then removed, and cells were resuspended in 10 µL ampicillin containing M9 media with the corresponding carbon source as indicated. Furthermore, 1 µL of the resuspended cell culture was added to the center of a 1% M9 agarose pad supplemented with ampicillin and the same carbon source, which was prepared according to Wilmaerts et al (55). A coverslip was added to the top of the agarose, and the cassette was placed inside a humid incubation chamber (Tokai Hit, Incubation Systems for Microscopes, Japan) at 37°C for imaging. Time-lapse images were acquired as processed using the same microscope and software as described in ROS measurements section. Time-lapse images were taken every 5 minutes.

### ROS scavenging assay

The ROS scavenging assays were performed using bipyridyl and thiourea and following a previous method (38). In the assay, a combination of 2,2′-bipyridyl (0.28 mM) and thiourea (90 mM) was added to cultures and/or LB agar plates. Sampling and survival determination were kept the same with the Nutrient Shift Persistence Assay.

### H_2_O_2_ sensitivity tests

Steady-state cells (OD 0.6) grown on M9 glucose or glycerol were 1:5 diluted to fresh media in a 96-well imaging plate. Different concentrations of H_2_O_2_ were applied to the cell culture and incubated at 37°C for 20 minutes. The plate was then moved to the Infinite F200PRO plate reader (TECAN, Männedorf, Switzerland), and growth profiles were recorded automatically. Growth data between OD 0.2 and 0.6 were used to calculate the growth rate. The lag time required for resuming growth was determined following the published method (24).

### Statistical analysis

Statistical analysis was performed using Origin 2021 bonded with a Paired Comparison Plot application. Data are from three biological replicates, if applicable. Statistical results are shown either with lowercase letters (significance level 0.05), asterisks (*P < 0.05, **P < 0.001, ***P < 0.0001), or nothing (non-significant). The statistical lowercase letter has the same color as the data series and is depicted in the picture legend. If two groups share the same letter (e.g., “a”), they are not significantly different. If two groups have different letters (e.g., “a” and “b”), they are significantly different.

## Supplementary Material

Reviewer comments

## SUPPLEMENTAL MATERIAL

Supplemental Material msystems.01295-24-s0001.docx Figures S1 to S3, Tables S1 to S3, and plasmid sequences.

OPEN PEER REVIEW reviewer-comments.pdf An accounting of the reviewer comments and feedback.

ASM does not own the copyrights to Supplemental Material that may be linked to, or accessed through, an article. The authors have granted ASM a non-exclusive, world-wide license to publish the Supplemental Material files. Please contact the corresponding author directly for reuse.
