# Supplementary material for: The ability in managing reactive oxygen species affects Escherichia coli persistence to ampicillin after nutrient shifts
Source: mSystems. 2024 Oct 29;9(11):e01295-24. doi: 10.1128/msystems.01295-24 (PMC11575164; doi:10.1128/msystems.01295-24)
Supplement: Supplemental Material — Figures S1 to S3, Tables S1 to S3, and plasmid sequences. [file msystems.01295-24-s0001.docx]

# The Ability in Managing Reactive Oxygen Species Affects *Escherichia coli* Persistence to Ampicillin after Nutrient Shifts

Ruixue Zhang ^a^, Christopher Hartline ^a^, Fuzhong Zhang ^a, b, c, *^

^a^ Department of Energy, Environmental & Chemical Engineering, Washington University in St. Louis, St. Louis, Missouri, USA

^b^ Division of Biological and Biomedical Sciences, Washington University in St. Louis, Saint Louis, Missouri, USA

^c^ Institute of Materials Science and Engineering, Washington University in St. Louis, Saint Louis, Missouri, USA

*Address correspondence to Dr. Fuzhong Zhang, [fzhang@seas.wustl.edu](mailto:fzhang@seas.wustl.edu)

**Supplementary materials**

| 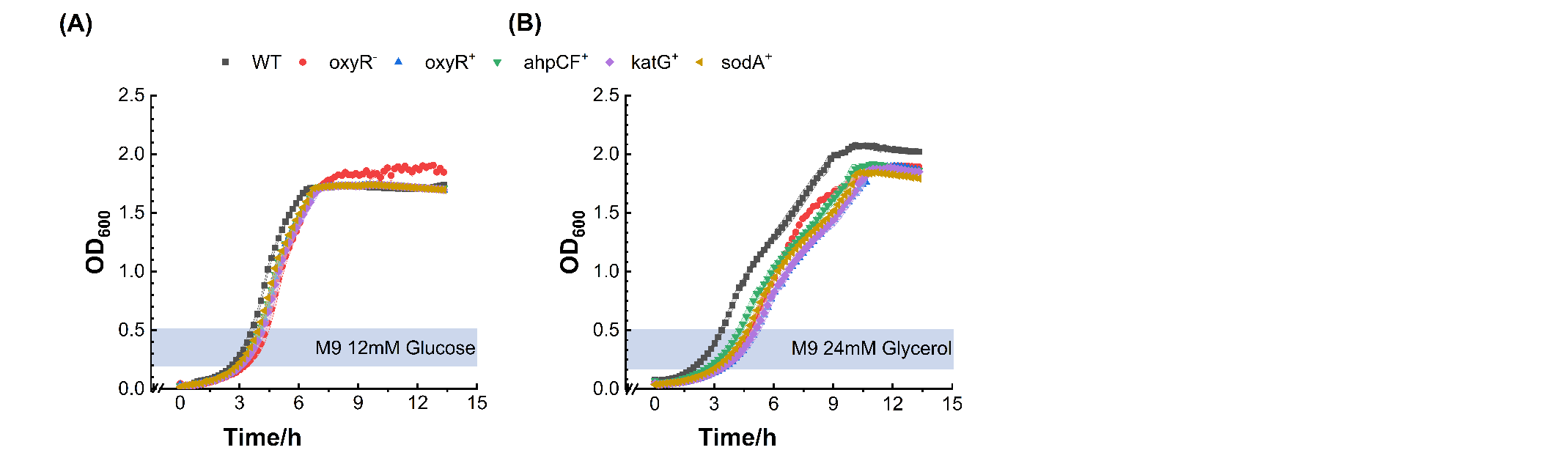 |
| --- |
| **Fig. S1 Pre-nutrient switch growth curves of strains used in this study.** Growth curves of *E. coli* growing in M9 with 12 mM glucose (A) or with 12 mM glycerol (B). |
|  |

| 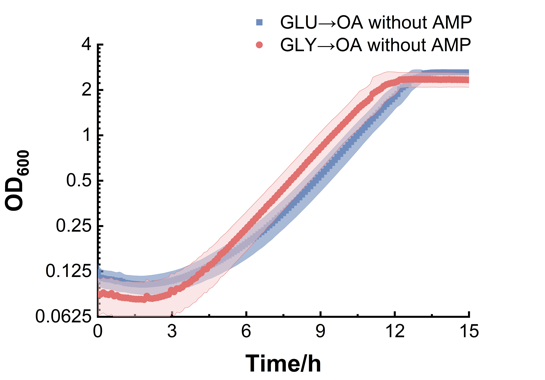 |
| --- |
| **Fig. S2 Cell growth after nutrient switch from glucose (blue) and glycerol (red) to OA without ampicillin** |

| **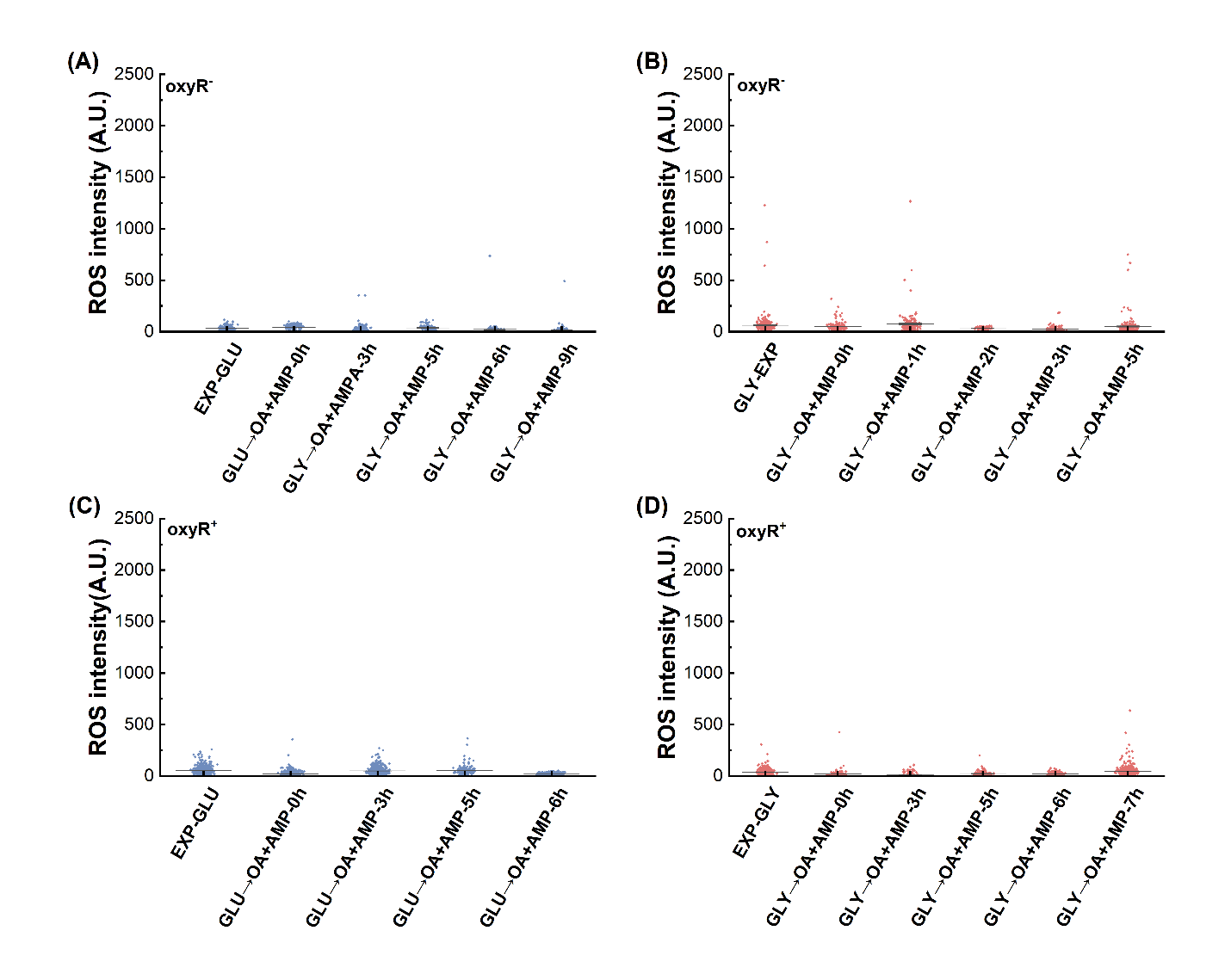** |
| --- |
| **Fig. S3** **Relative** **ROS intensity of single cells (n>300 from biological triplicates) at different time points after nutrient shift.** Results of *oxyR^-^* mutant cells after nutrient switch from glucose (A) or glycerol (B) to OA with ampicillin. Results of *oxyR^+^* mutant cells after switching from glucose (C) or glycerol (D) to OA with ampicillin. Statistical assessments were performed using a pairwise comparison with Holm-Sidak’s test and none of them are significant different from the rest. |

| **Table S1 Pre-switch growth rates (h^-1^) calculated between OD600 0.2~0.5** | | |
| --- | --- | --- |
|  | **M9 12mM Glucose** | **M9 24mM Glycerol** |
| **WT** | 0.89±0.07 | 0.71±0.00 |
| ***oxyR^-^*** | 0.88±0.05 | 0.71±0.01 |
| ***oxyR^+^*** | 0.89±0.01 | 0.62±0.00 |
| ***ahpCF^+^*** | 0.88±0.02 | 0.63±0.00 |
| ***katG^+^*** | 0.88±0.01 | 0.62±0.00 |
| ***sodA^+^*** | 0.88±0.01 | 0.63±0.00 |

# Table S2 Strains

| **Strain** | **Genotype** | **Source or Reference** |
| --- | --- | --- |
| NCM3722 (WT) | F^+^ | CGSC#:12355 |
| MDS42pdu | MDS42 with polB， dinB， umuDC deleted | (1) |
| FadD-YFP Strain | NCM3722 φ(*fadD-YFP*) | (2) |
| NCM3722 *oxyR*^+^ | NCM3722 with ps6c-oxyR | This work |
| NCM3722 *oxyR*^-^ | NCM3722 with oxyR deleted | This work |
| NCM3722 *katG*^+^ | NCM3722 with ps6c-katG | This work |
| NCM3722 *sodA*^+^ | NCM3722 with ps6c-sodA | This work |
| NCM3722 *ahpCF*^+^ | NCM3722 with ps6c-ahpCF | This work |
| NCM3722 *gfp^+^* | NCM3722 with ps6c-gfp | This work |

# Table S3 Plasmids

| **Plasmid** | **Function** | **Source** | **Sequence** |
| --- | --- | --- | --- |
| pTarget_delta oxyR | Used for knock-out of oxidative stress regulaor OxyR from genome | This work | Attached below |
| ps6c-oxyR | Used for overexpression of oxidative stress regulator oxyR when induced with 1mM IPTG | This work |  |
| ps6c-katG | Used for overexpression of ROS detoxification enzyme katG when induced with 1mM IPTG | This work |  |
| ps6c-sodA | Used for overexpression of ROS detoxification enzyme sodA when induced with 1mM IPTG | This work |  |
| ps6c-ahpCF | Used for overexpression of ROS detoxification enzymes ahpC and ahpF when induced with 1mM IPTG | This work |  |
| ps6c-GFP | Used for overexpression of GFP when induced with 1mM IPTG | This work |  |

**pTarget_delta oxyR:**

ctcgagttcatgtgcagctccatcagcaaaaggggatgataagtttatcaccaccgactatttgcaacagtgccgttgatcgtgctatgatcgactgatgtcatcagcggtggagtgcaatgtcatgagggaagcggtgatcgccgaagtatcgactcaactatcagaggtagttggcgtcatcgagcgccatctcgaaccgacgttgctggccgtacatttgtacggctccgcagtggatggcggcctgaagccacacagtgatattgatttgctggttacggtgaccgtaaggcttgatgaaacaacgcggcgagctttgatcaacgaccttttggaaacttcggcttcccctggagagagcgagattctccgcgctgtagaagtcaccattgttgtgcacgacgacatcattccgtggcgttatccagctaagcgcgaactgcaatttggagaatggcagcgcaatgacattcttgcaggtatcttcgagccagccacgatcgacattgatctggctatcttgctgacaaaagcaagagaacatagcgttgccttggtaggtccagcggcggaggaactctttgatccggttcctgaacaggatctatttgaggcgctaaatgaaaccttaacgctatggaactcgccgcccgactgggctggcgatgagcgaaatgtagtgcttacgttgtcccgcatttggtacagcgcagtaaccggcaaaatcgcgccgaaggatgtcgctgccgactgggcaatggagcgcctgccggcccagtatcagcccgtcatacttgaagctagacaggcttatcttggacaagaagaagatcgcttggcctcgcgcgcagatcagttggaagaatttgtccactacgtgaaaggcgagatcaccaaggtagtcggcaaataagatgccgctcgccagtcgattggctgagctcatgaagttcctattccgaagttccgcgaacgcgtaaaggatctaggtgaagatcctttttgataatctcatgaccaaaatcccttaacgtgagttttcgttccactgagcgtcagaccccgtagaaaagatcaaaggatcttcttgagatcctttttttctgcgcgtaatctgctgcttgcaaacaaaaaaaccaccgctaccagcggtggtttgtttgccggatcaagagctaccaactctttttccgaaggtaactggcttcagcagagcgcagataccaaatactgtccttctagtgtagccgtagttaggccaccacttcaagaactctgtagcaccgcctacatacctcgctctgctaatcctgttaccagtggctgctgccagtggcgataagtcgtgtcttaccgggttggactcaagacgatagttaccggataaggcgcagcggtcgggctgaacggggggttcgtgcacacagcccagcttggagcgaacgacctacaccgaactgagatacctacagcgtgagctatgagaaagcgccacgcttcccgaagggagaaaggcggacaggtatccggtaagcggcagggtcggaacaggagagcgcacgagggagcttccagggggaaacgcctggtatctttatagtcctgtcgggtttcgccacctctgacttgagcgtcgatttttgtgatgctcgtcaggggggcggagcctatggaaaaacgccagcaacgcggcctttttacggttcctggccttttgctggccttttgctcacatgttctttcctgcgttatcccctgattctgtggataaccgtattaccgcctttgagtgagctgataccgctcgccgcagccgaacgaccgagcgcagcgagtcagtgagcgaggaagcggaagagcgcctgatgcggtattttctccttacgcatctgtgcggtatttcacaccgcatatgctggatccttgacagctagctcagtcctaggtataatactagtgtgtgtatcttcatccgcccgttttagagctagaaatagcaagttaaaataaggctagtccgttatcaacttgaaaaagtggcaccgagtcggtgctttttttgaattctctagagtcgacctgcagaagcttagatctcgcaccatattgttggtgaagcggtggtggaagccattcgtcagggcaaaccgctggaagatctgccgctcagtgagttgcagaaattcagtcaggtgattgacgaagatgtctatccgattctgtcgctgcaatcgtgcctcgacaagcgtgcggcaaaaggcggcgtctcaccgcagcaggtggcgcaggcgattgcttttgcgcaggctcggttagggtaagaacatttatatgtataaatttgagcctggcttatcgccgggcttttttatggcaaaaaaaagcggatcctggagatccgcaaaagttcacgttggctttagttattcgagttgagaaactctcgaaacgggcagtgacttcaagggttaaaagaggtgccgctccgtttctgtgagcaattatcagtcagaatgcttgatagggataatcgttcattgctattctacctatcgccatgaactatcgtggcgatggcagaggccatccgcgcaagaatggatggccatttcgataaagttttaaaacaggcggtttaaaccgtttaacgcagctacccgataggcttccgccatcgtcgggtagttaaaggtggtgttgacgaagtactcaatagtgttgccgccacctttctgttccataatcgcctgaccgatatgaataatttcggcagcgcgctcgccaaagcagtgaatacccagaatctcttttgtttcccgatggaacaaaattttcagcgtgcccacgttcatgccgacgatttgtgcgcgtgccagatgtttaaactgggcgcggcccacttcatatggcactttcattgcggtcagctgctgttcggttttgcccacagagctgatttccgggatggtgtaaataccggtagggatatcttcaatcagatgtgcggtggcttcgccttttaccagcgcctgcgcggcaatgcgcccctggtcataggccgccgacgccaggctcggataaccaatcacgtcgcccaccgcgtaaacgtgtggctgtgcggtctgatacatgctgttgaccttcagctgtccgcggctgtcagtttctagcccaatgttctgtaacgccagcgaatcggtattaccggtgcgaccgttggcatagagcaggc

**ps6c-oxyR:**

ggatccaaactcgagtaaggatctccaggcatcaaataaaacgaaaggctcagtcgaaagactgggcctttcgttttatctgttgtttgtcggtgaacgctctctactagagtcacactggctcaccttcgggtgggcctttctgcgtttatacctagggtacgggttttgctgcccgcaaacgggctgttctggtgttgctagtttgttatcagaatcgcagatccggcttcagccggtttgccggctgaaagcgctatttcttccagaattgccatgattttttccccacgggaggcgtcactggctcccgtgttgtcggcagctttgattcgataagcagcatcgcctgtttcaggctgtctatgtgtgactgttgagctgtaacaagttgtctcaggtgttcaatttcatgttctagttgctttgttttactggtttcacctgttctattaggtgttacatgctgttcatctgttacattgtcgatctgttcatggtgaacagctttgaatgcaccaaaaactcgtaaaagctctgatgtatctatcttttttacaccgttttcatctgtgcatatggacagttttccctttgatatgtaacggtgaacagttgttctacttttgtttgttagtcttgatgcttcactgatagatacaagagccataagaacctcagatccttccgtatttagccagtatgttctctagtgtggttcgttgtttttgcgtgagccatgagaacgaaccattgagatcatacttactttgcatgtcactcaaaaattttgcctcaaaactggtgagctgaatttttgcagttaaagcatcgtgtagtgtttttcttagtccgttatgtaggtaggaatctgatgtaatggttgttggtattttgtcaccattcatttttatctggttgttctcaagttcggttacgagatccatttgtctatctagttcaacttggaaaatcaacgtatcagtcgggcggcctcgcttatcaaccaccaatttcatattgctgtaagtgtttaaatctttacttattggtttcaaaacccattggttaagccttttaaactcatggtagttattttcaagcattaacatgaacttaaattcatcaaggctaatctctatatttgccttgtgagttttcttttgtgttagttcttttaataaccactcataaatcctcatagagtatttgttttcaaaagacttaacatgttccagattatattttatgaatttttttaactggaaaagataaggcaatatctcttcactaaaaactaattctaatttttcgcttgagaacttggcatagtttgtccactggaaaatctcaaagcctttaaccaaaggattcctgatttccacagttctcgtcatcagctctctggttgctttagctaatacaccataagcattttccctactgatgttcatcatctgagcgtattggttataagtgaacgataccgtccgttctttccttgtagggttttcaatcgtggggttgagtagtgccacacagcataaaattagcttggtttcatgctccgttaagtcatagcgactaatcgctagttcatttgctttgaaaacaactaattcagacatacatctcaattggtctaggtgattttaatcactataccaattgagatgggctagtcaatgataattactagtccttttcccgggtgatctgggtatctgtaaattctgctagacctttgctggaaaacttgtaaattctgctagaccctctgtaaattccgctagacctttgtgtgttttttttgtttatattcaagtggttataatttatagaataaagaaagaataaaaaaagataaaaagaatagatcccagccctgtgtataactcactactttagtcagttccgcagtattacaaaaggatgtcgcaaacgctgtttgctcctctacaaaacagaccttaaaaccctaaaggcttaagtagcaccctcgcaagctcgggcaaatcgctgaatattccttttgtctccgaccatcaggcacctgagtcgctgtctttttcgtgacattcagttcgctgcgctcacggctctggcagtgaatgggggtaaatggcactacaggcgccttttatggattcatgcaaggaaactacccataatacaagaaaagcccgtcacgggcttctcagggcgttttatggcgggtctgctatgtggtgctatctgactttttgctgttcagcagttcctgccctctgattttccagtctgaccacttcggattatcccgtgacaggtcattcagactggctaatgcacccagtaaggcagcggtatcatcaacaggcttacccgtcttactgtccctagtgcttggattctcaccaataaaaaacgcccggcggcaaccgagcgttctgaacaaatccagatggagttctgaggtcattactggatctatcaacaggagtccaagcgagctcgatatcaaattacgccccgccctgccactcatcgcagtactgttgtaattcattaagcattctgccgacatggaagccatcacaaacggcatgatgaacctgaatcgccagcggcatcagcaccttgtcgccttgcgtataatatttgcccatggtgaaaacgggggcgaagaagttgtccatattggccacgtttaaatcaaaactggtgaaactcacccagggattggctgagacgaaaaacatattctcaataaaccctttagggaaataggccaggttttcaccgtaacacgccacatcttgcgaatatatgtgtagaaactgccggaaatcgtcgtggtattcactccagagcgatgaaaacgtttcagtttgctcatggaaaacggtgtaacaagggtgaacactatcccatatcaccagctcaccgtctttcattgccatacgaaattccggatgagcattcatcaggcgggcaagaatgtgaataaaggccggataaaacttgtgcttatttttctttacggtctttaaaaaggccgtaatatccagctgaacggtctggttataggtacattgagcaactgactgaaatgcctcaaaatgttctttacgatgccattgggatatatcaacggtggtatatccagtgatttttttctccattttagcttccttagctcctgaaaatctcgataactcaaaaaatacgcccggtagtgatcttatttcattatggtgaaagttggaacctcttacgtgccgatcaacgtctcattttcgccagatatcgacgtcggtgcctaatgagtgagctaacttacattaattgcgttgcgctcactgcccgctttccagtcgggaaacctgtcgtgccagctgcattaatgaatcggccaacgcgcggggagaggcggtttgcgtattgggcgccagggtggtttttcttttcaccagtgagacgggcaacagctgattgcccttcaccgcctggccctgagagagttgcagcaagcggtccacgctggtttgccccagcaggcgaaaatcctgtttgatggtggttaacggcgggatataacatgagctgtcttcggtatcgtcgtatcccactaccgagatgtccgcaccaacgcgcagcccggactcggtaatggcgcgcattgcgcccagcgccatctgatcgttggcaaccagcatcgcagtgggaacgatgccctcattcagcatttgcatggtttgttgaaaaccggacatggcactccagtcgccttcccgttccgctatcggctgaatttgattgcgagtgagatatttatgccagccagccagacgcagacgcgccgagacagaacttaatgggcccgctaacagcgcgatttgctggtgacccaatgcgaccagatgctccacgcccagtcgcgtaccgtcttcatgggagaaaataatactgttgatgggtgtctggtcagagacatcaagaaataacgccggaacattagtgcaggcagcttccacagcaatggcatcctggtcatccagcggatagttaatgatcagcccactgacgcgttgcgcgagaagattgtgcaccgccgctttacaggcttcgacgccgcttcgttctaccatcgacaccaccacgctggcacccagttgatcggcgcgagatttaatcgccgcgacaatttgcgacggcgcgtgcagggccagactggaggtggcaacgccaatcagcaacgactgtttgcccgccagttgttgtgccacgcggttgggaatgtaattcagctccgccatcgccgcttccactttttcccgcgttttcgcagaaacgtggctggcctggttcaccacgcgggaaacggtctgataagagacaccggcatactctgcgacatcgtataacgttactggtttcacattcaccaccctgaattgactctcttccgggcgctatcatgccataccgcgaaaggttttgcgccattcgatggtgtccgggatctcgacgctctcccttatgcgactcctgcattaggaagcagcccagtagtaggttgaggccgttgagcaccgccgccgcaaggaatggtgcatgcaaggagatggcgcccaacagtcccccggccacggggcctgccaccatacccacgccgaaacaagcgctcatgagcccgaagtggcgagcccgatcttccccatcggtgatgtcggcgatataggcgccagcaaccgcacctgtggcgccggtgatgccggccacgatgcgtccggcgtagaggatcgagaattgtgagcggataacaattgacattgtgagcggataacaagatactgagcacatcagcaggacgcactgaccgaattcaaaagatctgatacctctccctgggaagagaatatgaatattcgtgatcttgagtacctggtggcattggctgaacaccgccattttcggcgtgcggcagattcctgccacgttagccagccgacgcttagcgggcaaattcgtaagctggaagatgagctgggcgtgatgttgctggagcggaccagccgtaaagtgttgttcacccaggcgggaatgctgctggtggatcaggcgcgtaccgtgctgcgtgaggtgaaagtccttaaagagatggcaagccagcagggcgagacgatgtccggaccgctgcacattggtttgattcccacagttggaccgtacctgctaccgcatattatccctatgctgcaccagacctttccaaagctggaaatgtatctgcatgaagcacagacccaccagttactggcgcaactggacagcggcaaactcgattgcgtgatcctcgcgctggtgaaagagagcgaagcattcattgaagtgccgttgtttgatgagccaatgttgctggctatctatgaagatcacccgtgggcgaaccgcgaatgcgtaccgatggccgatctggcaggggaaaaactgctgatgctggaagatggtcactgtttgcgcgatcaggcaatgggtttctgttttgaagccggggcggatgaagatacacacttccgcgcgaccagcctggaaactctgcgcaacatggtggcggcaggtagcgggatcactttactgccagcgctggctgtgccgccggagcgcaaacgcgatggggttgtttatctgccgtgcattaagccggaaccacgccgcactattggcctggtttatcgtcctggctcaccgctgcgcagccgctatgagcagctggcagaggccatccgcgcaagaatggatggccatttcgataaagttttaaaacaggcggtttaa

**ps6c_katG:**

ggatccaaactcgagtaaggatctccaggcatcaaataaaacgaaaggctcagtcgaaagactgggcctttcgttttatctgttgtttgtcggtgaacgctctctactagagtcacactggctcaccttcgggtgggcctttctgcgtttatacctagggtacgggttttgctgcccgcaaacgggctgttctggtgttgctagtttgttatcagaatcgcagatccggcttcagccggtttgccggctgaaagcgctatttcttccagaattgccatgattttttccccacgggaggcgtcactggctcccgtgttgtcggcagctttgattcgataagcagcatcgcctgtttcaggctgtctatgtgtgactgttgagctgtaacaagttgtctcaggtgttcaatttcatgttctagttgctttgttttactggtttcacctgttctattaggtgttacatgctgttcatctgttacattgtcgatctgttcatggtgaacagctttgaatgcaccaaaaactcgtaaaagctctgatgtatctatcttttttacaccgttttcatctgtgcatatggacagttttccctttgatatgtaacggtgaacagttgttctacttttgtttgttagtcttgatgcttcactgatagatacaagagccataagaacctcagatccttccgtatttagccagtatgttctctagtgtggttcgttgtttttgcgtgagccatgagaacgaaccattgagatcatacttactttgcatgtcactcaaaaattttgcctcaaaactggtgagctgaatttttgcagttaaagcatcgtgtagtgtttttcttagtccgttatgtaggtaggaatctgatgtaatggttgttggtattttgtcaccattcatttttatctggttgttctcaagttcggttacgagatccatttgtctatctagttcaacttggaaaatcaacgtatcagtcgggcggcctcgcttatcaaccaccaatttcatattgctgtaagtgtttaaatctttacttattggtttcaaaacccattggttaagccttttaaactcatggtagttattttcaagcattaacatgaacttaaattcatcaaggctaatctctatatttgccttgtgagttttcttttgtgttagttcttttaataaccactcataaatcctcatagagtatttgttttcaaaagacttaacatgttccagattatattttatgaatttttttaactggaaaagataaggcaatatctcttcactaaaaactaattctaatttttcgcttgagaacttggcatagtttgtccactggaaaatctcaaagcctttaaccaaaggattcctgatttccacagttctcgtcatcagctctctggttgctttagctaatacaccataagcattttccctactgatgttcatcatctgagcgtattggttataagtgaacgataccgtccgttctttccttgtagggttttcaatcgtggggttgagtagtgccacacagcataaaattagcttggtttcatgctccgttaagtcatagcgactaatcgctagttcatttgctttgaaaacaactaattcagacatacatctcaattggtctaggtgattttaatcactataccaattgagatgggctagtcaatgataattactagtccttttcccgggtgatctgggtatctgtaaattctgctagacctttgctggaaaacttgtaaattctgctagaccctctgtaaattccgctagacctttgtgtgttttttttgtttatattcaagtggttataatttatagaataaagaaagaataaaaaaagataaaaagaatagatcccagccctgtgtataactcactactttagtcagttccgcagtattacaaaaggatgtcgcaaacgctgtttgctcctctacaaaacagaccttaaaaccctaaaggcttaagtagcaccctcgcaagctcgggcaaatcgctgaatattccttttgtctccgaccatcaggcacctgagtcgctgtctttttcgtgacattcagttcgctgcgctcacggctctggcagtgaatgggggtaaatggcactacaggcgccttttatggattcatgcaaggaaactacccataatacaagaaaagcccgtcacgggcttctcagggcgttttatggcgggtctgctatgtggtgctatctgactttttgctgttcagcagttcctgccctctgattttccagtctgaccacttcggattatcccgtgacaggtcattcagactggctaatgcacccagtaaggcagcggtatcatcaacaggcttacccgtcttactgtccctagtgcttggattctcaccaataaaaaacgcccggcggcaaccgagcgttctgaacaaatccagatggagttctgaggtcattactggatctatcaacaggagtccaagcgagctcgatatcaaattacgccccgccctgccactcatcgcagtactgttgtaattcattaagcattctgccgacatggaagccatcacaaacggcatgatgaacctgaatcgccagcggcatcagcaccttgtcgccttgcgtataatatttgcccatggtgaaaacgggggcgaagaagttgtccatattggccacgtttaaatcaaaactggtgaaactcacccagggattggctgagacgaaaaacatattctcaataaaccctttagggaaataggccaggttttcaccgtaacacgccacatcttgcgaatatatgtgtagaaactgccggaaatcgtcgtggtattcactccagagcgatgaaaacgtttcagtttgctcatggaaaacggtgtaacaagggtgaacactatcccatatcaccagctcaccgtctttcattgccatacgaaattccggatgagcattcatcaggcgggcaagaatgtgaataaaggccggataaaacttgtgcttatttttctttacggtctttaaaaaggccgtaatatccagctgaacggtctggttataggtacattgagcaactgactgaaatgcctcaaaatgttctttacgatgccattgggatatatcaacggtggtatatccagtgatttttttctccattttagcttccttagctcctgaaaatctcgataactcaaaaaatacgcccggtagtgatcttatttcattatggtgaaagttggaacctcttacgtgccgatcaacgtctcattttcgccagatatcgacgtcggtgcctaatgagtgagctaacttacattaattgcgttgcgctcactgcccgctttccagtcgggaaacctgtcgtgccagctgcattaatgaatcggccaacgcgcggggagaggcggtttgcgtattgggcgccagggtggtttttcttttcaccagtgagacgggcaacagctgattgcccttcaccgcctggccctgagagagttgcagcaagcggtccacgctggtttgccccagcaggcgaaaatcctgtttgatggtggttaacggcgggatataacatgagctgtcttcggtatcgtcgtatcccactaccgagatgtccgcaccaacgcgcagcccggactcggtaatggcgcgcattgcgcccagcgccatctgatcgttggcaaccagcatcgcagtgggaacgatgccctcattcagcatttgcatggtttgttgaaaaccggacatggcactccagtcgccttcccgttccgctatcggctgaatttgattgcgagtgagatatttatgccagccagccagacgcagacgcgccgagacagaacttaatgggcccgctaacagcgcgatttgctggtgacccaatgcgaccagatgctccacgcccagtcgcgtaccgtcttcatgggagaaaataatactgttgatgggtgtctggtcagagacatcaagaaataacgccggaacattagtgcaggcagcttccacagcaatggcatcctggtcatccagcggatagttaatgatcagcccactgacgcgttgcgcgagaagattgtgcaccgccgctttacaggcttcgacgccgcttcgttctaccatcgacaccaccacgctggcacccagttgatcggcgcgagatttaatcgccgcgacaatttgcgacggcgcgtgcagggccagactggaggtggcaacgccaatcagcaacgactgtttgcccgccagttgttgtgccacgcggttgggaatgtaattcagctccgccatcgccgcttccactttttcccgcgttttcgcagaaacgtggctggcctggttcaccacgcgggaaacggtctgataagagacaccggcatactctgcgacatcgtataacgttactggtttcacattcaccaccctgaattgactctcttccgggcgctatcatgccataccgcgaaaggttttgcgccattcgatggtgtccgggatctcgacgctctcccttatgcgactcctgcattaggaagcagcccagtagtaggttgaggccgttgagcaccgccgccgcaaggaatggtgcatgcaaggagatggcgcccaacagtcccccggccacggggcctgccaccatacccacgccgaaacaagcgctcatgagcccgaagtggcgagcccgatcttccccatcggtgatgtcggcgatataggcgccagcaaccgcacctgtggcgccggtgatgccggccacgatgcgtccggcgtagaggatcgagaattgtgagcggataacaattgacattgtgagcggataacaagatactgagcacatcagcaggacgcactgaccgaattcaaaagatctgatacctctccctgggaagagaatatgagcacgtcagacgatatccataacaccacagccactggcaaatgcccgttccatcagggcggtcacgaccagagtgcgggggcgggcacaaccactcgcgactggtggccaaatcaacttcgtgttgacctgttaaaccaacattctaatcgttctaacccactgggtgaggactttgactaccgcaaagaattcagcaaattagattactacggcctgaaaaaagatctgaaagccctgttgacagaatctcaaccgtggtggccagccgactggggcagttacgccggtctgtttattcgtatggcctggcacggcgcggggacttaccgttcaatcgatggacgcggtggcgcgggtcgtggtcagcaacgttttgcaccgctgaactcctggccggataacgtaagcctcgataaagcgcgtcgcctgttgtggccaatcaaacagaaatatggtcagaaaatctcctgggccgacctgtttatcctcgcgggtaacgtggcgctagaaaactccggcttccgtaccttcggttttggtgccggtcgtgaagacgtctgggaaccggatctggatgttaactggggtgatgaaaaagcctggctgactcaccgtcatccggaagcgctggcgaaagcaccgctgggtgcaaccgagatgggtctgatttacgttaacccggaaggcccggatcacagcggcgaaccgctttctgcggcagcagctatccgcgcgaccttcggcaacatgggcatgaacgacgaagaaaccgtggcgctgattgcgggtggtcatacgctgggtaaaacccacggtgccggtccgacatcaaatgtaggtcctgatccagaagctgcaccgattgaagaacaaggtttaggttgggcgagcacttacggcagcggcgttggcgcagatgccattacctctggtctggaagtagtctggacccagacgccgacccagtggagcaactatttcttcgagaacctgttcaagtatgagtgggtacagacccgcagcccggctggcgcaatccagttcgaagcggtagacgcaccggaaattatcccggatccgtttgatccgtcgaagaaacgtaaaccgacaatgctggtgaccgacctgacgctgcgttttgatcctgagttcgagaagatctctcgtcgtttcctcaacgatccgcaggcgttcaacgaagcctttgcccgtgcctggttcaaactgacgcacagggatatggggccgaaatctcgctacatcgggccggaagtgccgaaagaagatctgatctggcaagatccgctgccgcagccgatctacaacccgaccgagcaggacattatcgatctgaaattcgcgattgcggattctggtctgtctgttagtgagctggtatcggtggcctgggcatctgcttctaccttccgtggtggcgacaaacgcggtggtgccaacggtgcgcgtctggcattaatgccgcagcgcgactgggatgtgaacgccgcagccgttcgtgctctgcctgttctggagaaaatccagaaagagtctggtaaagcctcgctggcggatatcatagtgctggctggtgtggttggtgttgagaaagccgcaagcgccgcaggtttgagcattcatgtaccgtttgcgccgggtcgcgttgatgcgcgtcaggatcagactgacattgagatgtttgagctgctggagccaattgctgacggtttccgtaactatcgcgctcgtctggacgtttccaccaccgagtcactgctgatcgacaaagcacagcaactgacgctgaccgcgccggaaatgactgcgctggtgggcggcatgcgtgtactgggtgccaacttcgatggcagcaaaaacggcgtcttcactgaccgcgttggcgtattgagcaatgacttcttcgtgaacttgctggatatgcgttacgagtggaaagcgaccgacgaatcgaaagagctgttcgaaggccgtgaccgtgaaaccggcgaagtgaaatttacggccagccgtgcggatctggtgtttggttctaactccgtcctgcgtgcggtggcggaagtttacgccagtagcgatgcccacgagaagtttgttaaagacttcgtggcggcatgggtgaaagtgatgaacctcgaccgtttcgacctgctgtaa

**ps6c_sodA:**

ggatccaaactcgagtaaggatctccaggcatcaaataaaacgaaaggctcagtcgaaagactgggcctttcgttttatctgttgtttgtcggtgaacgctctctactagagtcacactggctcaccttcgggtgggcctttctgcgtttatacctagggtacgggttttgctgcccgcaaacgggctgttctggtgttgctagtttgttatcagaatcgcagatccggcttcagccggtttgccggctgaaagcgctatttcttccagaattgccatgattttttccccacgggaggcgtcactggctcccgtgttgtcggcagctttgattcgataagcagcatcgcctgtttcaggctgtctatgtgtgactgttgagctgtaacaagttgtctcaggtgttcaatttcatgttctagttgctttgttttactggtttcacctgttctattaggtgttacatgctgttcatctgttacattgtcgatctgttcatggtgaacagctttgaatgcaccaaaaactcgtaaaagctctgatgtatctatcttttttacaccgttttcatctgtgcatatggacagttttccctttgatatgtaacggtgaacagttgttctacttttgtttgttagtcttgatgcttcactgatagatacaagagccataagaacctcagatccttccgtatttagccagtatgttctctagtgtggttcgttgtttttgcgtgagccatgagaacgaaccattgagatcatacttactttgcatgtcactcaaaaattttgcctcaaaactggtgagctgaatttttgcagttaaagcatcgtgtagtgtttttcttagtccgttatgtaggtaggaatctgatgtaatggttgttggtattttgtcaccattcatttttatctggttgttctcaagttcggttacgagatccatttgtctatctagttcaacttggaaaatcaacgtatcagtcgggcggcctcgcttatcaaccaccaatttcatattgctgtaagtgtttaaatctttacttattggtttcaaaacccattggttaagccttttaaactcatggtagttattttcaagcattaacatgaacttaaattcatcaaggctaatctctatatttgccttgtgagttttcttttgtgttagttcttttaataaccactcataaatcctcatagagtatttgttttcaaaagacttaacatgttccagattatattttatgaatttttttaactggaaaagataaggcaatatctcttcactaaaaactaattctaatttttcgcttgagaacttggcatagtttgtccactggaaaatctcaaagcctttaaccaaaggattcctgatttccacagttctcgtcatcagctctctggttgctttagctaatacaccataagcattttccctactgatgttcatcatctgagcgtattggttataagtgaacgataccgtccgttctttccttgtagggttttcaatcgtggggttgagtagtgccacacagcataaaattagcttggtttcatgctccgttaagtcatagcgactaatcgctagttcatttgctttgaaaacaactaattcagacatacatctcaattggtctaggtgattttaatcactataccaattgagatgggctagtcaatgataattactagtccttttcccgggtgatctgggtatctgtaaattctgctagacctttgctggaaaacttgtaaattctgctagaccctctgtaaattccgctagacctttgtgtgttttttttgtttatattcaagtggttataatttatagaataaagaaagaataaaaaaagataaaaagaatagatcccagccctgtgtataactcactactttagtcagttccgcagtattacaaaaggatgtcgcaaacgctgtttgctcctctacaaaacagaccttaaaaccctaaaggcttaagtagcaccctcgcaagctcgggcaaatcgctgaatattccttttgtctccgaccatcaggcacctgagtcgctgtctttttcgtgacattcagttcgctgcgctcacggctctggcagtgaatgggggtaaatggcactacaggcgccttttatggattcatgcaaggaaactacccataatacaagaaaagcccgtcacgggcttctcagggcgttttatggcgggtctgctatgtggtgctatctgactttttgctgttcagcagttcctgccctctgattttccagtctgaccacttcggattatcccgtgacaggtcattcagactggctaatgcacccagtaaggcagcggtatcatcaacaggcttacccgtcttactgtccctagtgcttggattctcaccaataaaaaacgcccggcggcaaccgagcgttctgaacaaatccagatggagttctgaggtcattactggatctatcaacaggagtccaagcgagctcgatatcaaattacgccccgccctgccactcatcgcagtactgttgtaattcattaagcattctgccgacatggaagccatcacaaacggcatgatgaacctgaatcgccagcggcatcagcaccttgtcgccttgcgtataatatttgcccatggtgaaaacgggggcgaagaagttgtccatattggccacgtttaaatcaaaactggtgaaactcacccagggattggctgagacgaaaaacatattctcaataaaccctttagggaaataggccaggttttcaccgtaacacgccacatcttgcgaatatatgtgtagaaactgccggaaatcgtcgtggtattcactccagagcgatgaaaacgtttcagtttgctcatggaaaacggtgtaacaagggtgaacactatcccatatcaccagctcaccgtctttcattgccatacgaaattccggatgagcattcatcaggcgggcaagaatgtgaataaaggccggataaaacttgtgcttatttttctttacggtctttaaaaaggccgtaatatccagctgaacggtctggttataggtacattgagcaactgactgaaatgcctcaaaatgttctttacgatgccattgggatatatcaacggtggtatatccagtgatttttttctccattttagcttccttagctcctgaaaatctcgataactcaaaaaatacgcccggtagtgatcttatttcattatggtgaaagttggaacctcttacgtgccgatcaacgtctcattttcgccagatatcgacgtcggtgcctaatgagtgagctaacttacattaattgcgttgcgctcactgcccgctttccagtcgggaaacctgtcgtgccagctgcattaatgaatcggccaacgcgcggggagaggcggtttgcgtattgggcgccagggtggtttttcttttcaccagtgagacgggcaacagctgattgcccttcaccgcctggccctgagagagttgcagcaagcggtccacgctggtttgccccagcaggcgaaaatcctgtttgatggtggttaacggcgggatataacatgagctgtcttcggtatcgtcgtatcccactaccgagatgtccgcaccaacgcgcagcccggactcggtaatggcgcgcattgcgcccagcgccatctgatcgttggcaaccagcatcgcagtgggaacgatgccctcattcagcatttgcatggtttgttgaaaaccggacatggcactccagtcgccttcccgttccgctatcggctgaatttgattgcgagtgagatatttatgccagccagccagacgcagacgcgccgagacagaacttaatgggcccgctaacagcgcgatttgctggtgacccaatgcgaccagatgctccacgcccagtcgcgtaccgtcttcatgggagaaaataatactgttgatgggtgtctggtcagagacatcaagaaataacgccggaacattagtgcaggcagcttccacagcaatggcatcctggtcatccagcggatagttaatgatcagcccactgacgcgttgcgcgagaagattgtgcaccgccgctttacaggcttcgacgccgcttcgttctaccatcgacaccaccacgctggcacccagttgatcggcgcgagatttaatcgccgcgacaatttgcgacggcgcgtgcagggccagactggaggtggcaacgccaatcagcaacgactgtttgcccgccagttgttgtgccacgcggttgggaatgtaattcagctccgccatcgccgcttccactttttcccgcgttttcgcagaaacgtggctggcctggttcaccacgcgggaaacggtctgataagagacaccggcatactctgcgacatcgtataacgttactggtttcacattcaccaccctgaattgactctcttccgggcgctatcatgccataccgcgaaaggttttgcgccattcgatggtgtccgggatctcgacgctctcccttatgcgactcctgcattaggaagcagcccagtagtaggttgaggccgttgagcaccgccgccgcaaggaatggtgcatgcaaggagatggcgcccaacagtcccccggccacggggcctgccaccatacccacgccgaaacaagcgctcatgagcccgaagtggcgagcccgatcttccccatcggtgatgtcggcgatataggcgccagcaaccgcacctgtggcgccggtgatgccggccacgatgcgtccggcgtagaggatcgagaattgtgagcggataacaattgacattgtgagcggataacaagatactgagcacatcagcaggacgcactgaccgaattcaaaagatctgatacctctccctgggaagagaatatgagctataccctgccatccctgccgtatgcttacgatgccctggaaccgcacttcgataagcagaccatggaaatccaccacaccaaacaccatcagacctacgtaaacaacgccaacgcggcgctggaaagcctgccagaatttgccaacctgccggttgaagagctgatcaccaaactggaccagctgccagcagacaagaaaaccgtactgcgcaacaacgctggcggtcacgctaaccacagcctgttctggaaaggtctgaaaaaaggcaccaccctgcagggtgacctgaaagcggctatcgaacgtgacttcggctccgttgataacttcaaagcagaatttgaaaaagcggcagcttcccgctttggttccggctgggcatggctggtgctgaaaggcgataaactggcggtggtttctactgctaaccaggattctccgctgatgggtgaagctatttctggcgcttccggcttcccgattatgggcctggatgtgtgggaacatgcttactacctgaaattccagaaccgccgtccggactacattaaagagttctggaacgtggtgaactgggacgaagcagcggcacgttttgcggcgaaaaaataa

**ps6c_ahpCF:**

ggatccaaactcgagtaaggatctccaggcatcaaataaaacgaaaggctcagtcgaaagactgggcctttcgttttatctgttgtttgtcggtgaacgctctctactagagtcacactggctcaccttcgggtgggcctttctgcgtttatacctagggtacgggttttgctgcccgcaaacgggctgttctggtgttgctagtttgttatcagaatcgcagatccggcttcagccggtttgccggctgaaagcgctatttcttccagaattgccatgattttttccccacgggaggcgtcactggctcccgtgttgtcggcagctttgattcgataagcagcatcgcctgtttcaggctgtctatgtgtgactgttgagctgtaacaagttgtctcaggtgttcaatttcatgttctagttgctttgttttactggtttcacctgttctattaggtgttacatgctgttcatctgttacattgtcgatctgttcatggtgaacagctttgaatgcaccaaaaactcgtaaaagctctgatgtatctatcttttttacaccgttttcatctgtgcatatggacagttttccctttgatatgtaacggtgaacagttgttctacttttgtttgttagtcttgatgcttcactgatagatacaagagccataagaacctcagatccttccgtatttagccagtatgttctctagtgtggttcgttgtttttgcgtgagccatgagaacgaaccattgagatcatacttactttgcatgtcactcaaaaattttgcctcaaaactggtgagctgaatttttgcagttaaagcatcgtgtagtgtttttcttagtccgttatgtaggtaggaatctgatgtaatggttgttggtattttgtcaccattcatttttatctggttgttctcaagttcggttacgagatccatttgtctatctagttcaacttggaaaatcaacgtatcagtcgggcggcctcgcttatcaaccaccaatttcatattgctgtaagtgtttaaatctttacttattggtttcaaaacccattggttaagccttttaaactcatggtagttattttcaagcattaacatgaacttaaattcatcaaggctaatctctatatttgccttgtgagttttcttttgtgttagttcttttaataaccactcataaatcctcatagagtatttgttttcaaaagacttaacatgttccagattatattttatgaatttttttaactggaaaagataaggcaatatctcttcactaaaaactaattctaatttttcgcttgagaacttggcatagtttgtccactggaaaatctcaaagcctttaaccaaaggattcctgatttccacagttctcgtcatcagctctctggttgctttagctaatacaccataagcattttccctactgatgttcatcatctgagcgtattggttataagtgaacgataccgtccgttctttccttgtagggttttcaatcgtggggttgagtagtgccacacagcataaaattagcttggtttcatgctccgttaagtcatagcgactaatcgctagttcatttgctttgaaaacaactaattcagacatacatctcaattggtctaggtgattttaatcactataccaattgagatgggctagtcaatgataattactagtccttttcccgggtgatctgggtatctgtaaattctgctagacctttgctggaaaacttgtaaattctgctagaccctctgtaaattccgctagacctttgtgtgttttttttgtttatattcaagtggttataatttatagaataaagaaagaataaaaaaagataaaaagaatagatcccagccctgtgtataactcactactttagtcagttccgcagtattacaaaaggatgtcgcaaacgctgtttgctcctctacaaaacagaccttaaaaccctaaaggcttaagtagcaccctcgcaagctcgggcaaatcgctgaatattccttttgtctccgaccatcaggcacctgagtcgctgtctttttcgtgacattcagttcgctgcgctcacggctctggcagtgaatgggggtaaatggcactacaggcgccttttatggattcatgcaaggaaactacccataatacaagaaaagcccgtcacgggcttctcagggcgttttatggcgggtctgctatgtggtgctatctgactttttgctgttcagcagttcctgccctctgattttccagtctgaccacttcggattatcccgtgacaggtcattcagactggctaatgcacccagtaaggcagcggtatcatcaacaggcttacccgtcttactgtccctagtgcttggattctcaccaataaaaaacgcccggcggcaaccgagcgttctgaacaaatccagatggagttctgaggtcattactggatctatcaacaggagtccaagcgagctcgatatcaaattacgccccgccctgccactcatcgcagtactgttgtaattcattaagcattctgccgacatggaagccatcacaaacggcatgatgaacctgaatcgccagcggcatcagcaccttgtcgccttgcgtataatatttgcccatggtgaaaacgggggcgaagaagttgtccatattggccacgtttaaatcaaaactggtgaaactcacccagggattggctgagacgaaaaacatattctcaataaaccctttagggaaataggccaggttttcaccgtaacacgccacatcttgcgaatatatgtgtagaaactgccggaaatcgtcgtggtattcactccagagcgatgaaaacgtttcagtttgctcatggaaaacggtgtaacaagggtgaacactatcccatatcaccagctcaccgtctttcattgccatacgaaattccggatgagcattcatcaggcgggcaagaatgtgaataaaggccggataaaacttgtgcttatttttctttacggtctttaaaaaggccgtaatatccagctgaacggtctggttataggtacattgagcaactgactgaaatgcctcaaaatgttctttacgatgccattgggatatatcaacggtggtatatccagtgatttttttctccattttagcttccttagctcctgaaaatctcgataactcaaaaaatacgcccggtagtgatcttatttcattatggtgaaagttggaacctcttacgtgccgatcaacgtctcattttcgccagatatcgacgtcggtgcctaatgagtgagctaacttacattaattgcgttgcgctcactgcccgctttccagtcgggaaacctgtcgtgccagctgcattaatgaatcggccaacgcgcggggagaggcggtttgcgtattgggcgccagggtggtttttcttttcaccagtgagacgggcaacagctgattgcccttcaccgcctggccctgagagagttgcagcaagcggtccacgctggtttgccccagcaggcgaaaatcctgtttgatggtggttaacggcgggatataacatgagctgtcttcggtatcgtcgtatcccactaccgagatgtccgcaccaacgcgcagcccggactcggtaatggcgcgcattgcgcccagcgccatctgatcgttggcaaccagcatcgcagtgggaacgatgccctcattcagcatttgcatggtttgttgaaaaccggacatggcactccagtcgccttcccgttccgctatcggctgaatttgattgcgagtgagatatttatgccagccagccagacgcagacgcgccgagacagaacttaatgggcccgctaacagcgcgatttgctggtgacccaatgcgaccagatgctccacgcccagtcgcgtaccgtcttcatgggagaaaataatactgttgatgggtgtctggtcagagacatcaagaaataacgccggaacattagtgcaggcagcttccacagcaatggcatcctggtcatccagcggatagttaatgatcagcccactgacgcgttgcgcgagaagattgtgcaccgccgctttacaggcttcgacgccgcttcgttctaccatcgacaccaccacgctggcacccagttgatcggcgcgagatttaatcgccgcgacaatttgcgacggcgcgtgcagggccagactggaggtggcaacgccaatcagcaacgactgtttgcccgccagttgttgtgccacgcggttgggaatgtaattcagctccgccatcgccgcttccactttttcccgcgttttcgcagaaacgtggctggcctggttcaccacgcgggaaacggtctgataagagacaccggcatactctgcgacatcgtataacgttactggtttcacattcaccaccctgaattgactctcttccgggcgctatcatgccataccgcgaaaggttttgcgccattcgatggtgtccgggatctcgacgctctcccttatgcgactcctgcattaggaagcagcccagtagtaggttgaggccgttgagcaccgccgccgcaaggaatggtgcatgcaaggagatggcgcccaacagtcccccggccacggggcctgccaccatacccacgccgaaacaagcgctcatgagcccgaagtggcgagcccgatcttccccatcggtgatgtcggcgatataggcgccagcaaccgcacctgtggcgccggtgatgccggccacgatgcgtccggcgtagaggatcgagaattgtgagcggataacaattgacattgtgagcggataacaagatactgagcacatcagcaggacgcactgaccgaattcaaaagatctgatacctctccctgggaagagaatatgtccttgattaacaccaaaattaaaccttttaaaaaccaggcattcaaaaacggcgaattcatcgaaatcaccgaaaaagataccgaaggccgctggagcgtcttcttcttctacccggctgactttactttcgtatgcccgaccgaactgggtgacgttgctgaccactacgaagaactgcagaaactgggcgtagacgtatacgcagtatctaccgatactcacttcacccacaaagcatggcacagcagctctgaaaccatcgctaaaatcaaatatgcgatgatcggcgacccgactggcgccctgacccgtaacttcgacaacatgcgtgaagatgaaggtctggctgaccgtgcgaccttcgttgttgacccgcagggtatcatccaggcaatcgaagttaccgctgaaggcattggccgtgacgcgtctgacctgctgcgtaaaatcaaagcagcacagtacgtagcttctcacccaggtgaagtttgcccggctaaatggaaagaaggtgaagcaactctggctccgtctctggacctggttggtaaaatctaaatttccttcgtctttcacgccatagcggcgttggcgtcgcccgctcaccccggtcacttacttgtgtaagctcccggggattcacaggctagccgccttgctctgacgcgaaatacttcggaaattcacctaattcttcgggtgctgcggcacccgattttcttccccgcaccatgatgcaagctgcatccaggtagccgcagaggccgcttgcatgatgatgtttaaagcccaggagataaacatgctcgacacaaatatgaaaactcaactcaaggcttaccttgagaaattgaccaagcctgttgagttaattgccacgctggatgacagcgctaaatcggcagaaatcaaggaactgttggctgaaatcgcagaactgtcagacaaagtcacctttaaagaagataacagcttgccggtgcgtaagccgtctttcctgatcaccaacccaggttccaaccaggggccacgttttgcaggctccccgctgggccacgagttcacctcgctggtactggcgttgctgtggaccggtggtcatccgtcgaaagaagcgcagtctctgctggagcagattcgccatattgacggtgattttgaattcgaaacctattactcgctctcttgccacaactgcccggacgtggtgcaggcgctgaacctgatgagcgtactgaacccgcgcatcaagcacactgcaattgacggcggcaccttccagaacgaaatcaccgatcgcaacgtgatgggcgttccggcagtgttcgtaaacgggaaagagtttggtcagggccgcatgacgttgactgaaatcgttgccaaaattgatactggcgcggaaaaacgtgcggcagaagagctgaacaagcgtgatgcttatgacgtattaatcgtcggttccggcccggcgggtgcagcggcagcaatttactccgcacgtaaaggcatccgtaccggtctgatgggcgaacgttttggtggtcagatcctcgataccgttgatatcgaaaactacatttctgtaccgaagactgaagggcagaagctggcaggcgcactgaaagttcacgttgatgaatacgacgttgatgtgatcgacagccagagcgccagcaaactgatcccagcagcagttgaaggtggtctgcatcagattgaaacagcttctggcgcggtactgaaagcacgcagcattatcgtggcgaccggtgcaaaatggcgcaacatgaacgttccgggcgaagatcagtatcgcaccaaaggcgtgacctactgcccgcactgcgacggcccgctgtttaaaggtaaacgcgtagcggttatcggcggcggtaactccggcgtggaagcggcaattgacctggcgggtatcgttgagcacgtaacgctgctggaatttgcgccagaaatgaaagccgaccaggttctgcaggacaaactgcgcagcctgaaaaacgtcgacattattctgaatgcgcaaaccacggaagtgaaaggcgacggcagcaaagtcgttggtctggaatatcgagatcgtgtcagcggcgatattcacaacatcgaactggccggtattttcgtccagattggtctgctgccgaacaccaactggctcgaaggcgcagtcgaacgtaaccgcatgggcgagattatcattgatgcgaaatgcgaaaccaacgtgaaaggcgtgttcgcagcgggtgactgtacgacggttccgtacaagcagatcatcatcgccactggcgaaggtgccaaagcctctctgagtgcttttgactacctgattcgcaccaaaactgcataa

ps6c_GFP:

ggatccaaactcgagtaaggatctccaggcatcaaataaaacgaaaggctcagtcgaaagactgggcctttcgttttatctgttgtttgtcggtgaacgctctctactagagtcacactggctcaccttcgggtgggcctttctgcgtttatacctagggtacgggttttgctgcccgcaaacgggctgttctggtgttgctagtttgttatcagaatcgcagatccggcttcagccggtttgccggctgaaagcgctatttcttccagaattgccatgattttttccccacgggaggcgtcactggctcccgtgttgtcggcagctttgattcgataagcagcatcgcctgtttcaggctgtctatgtgtgactgttgagctgtaacaagttgtctcaggtgttcaatttcatgttctagttgctttgttttactggtttcacctgttctattaggtgttacatgctgttcatctgttacattgtcgatctgttcatggtgaacagctttgaatgcaccaaaaactcgtaaaagctctgatgtatctatcttttttacaccgttttcatctgtgcatatggacagttttccctttgatatgtaacggtgaacagttgttctacttttgtttgttagtcttgatgcttcactgatagatacaagagccataagaacctcagatccttccgtatttagccagtatgttctctagtgtggttcgttgtttttgcgtgagccatgagaacgaaccattgagatcatacttactttgcatgtcactcaaaaattttgcctcaaaactggtgagctgaatttttgcagttaaagcatcgtgtagtgtttttcttagtccgttatgtaggtaggaatctgatgtaatggttgttggtattttgtcaccattcatttttatctggttgttctcaagttcggttacgagatccatttgtctatctagttcaacttggaaaatcaacgtatcagtcgggcggcctcgcttatcaaccaccaatttcatattgctgtaagtgtttaaatctttacttattggtttcaaaacccattggttaagccttttaaactcatggtagttattttcaagcattaacatgaacttaaattcatcaaggctaatctctatatttgccttgtgagttttcttttgtgttagttcttttaataaccactcataaatcctcatagagtatttgttttcaaaagacttaacatgttccagattatattttatgaatttttttaactggaaaagataaggcaatatctcttcactaaaaactaattctaatttttcgcttgagaacttggcatagtttgtccactggaaaatctcaaagcctttaaccaaaggattcctgatttccacagttctcgtcatcagctctctggttgctttagctaatacaccataagcattttccctactgatgttcatcatctgagcgtattggttataagtgaacgataccgtccgttctttccttgtagggttttcaatcgtggggttgagtagtgccacacagcataaaattagcttggtttcatgctccgttaagtcatagcgactaatcgctagttcatttgctttgaaaacaactaattcagacatacatctcaattggtctaggtgattttaatcactataccaattgagatgggctagtcaatgataattactagtccttttcccgggtgatctgggtatctgtaaattctgctagacctttgctggaaaacttgtaaattctgctagaccctctgtaaattccgctagacctttgtgtgttttttttgtttatattcaagtggttataatttatagaataaagaaagaataaaaaaagataaaaagaatagatcccagccctgtgtataactcactactttagtcagttccgcagtattacaaaaggatgtcgcaaacgctgtttgctcctctacaaaacagaccttaaaaccctaaaggcttaagtagcaccctcgcaagctcgggcaaatcgctgaatattccttttgtctccgaccatcaggcacctgagtcgctgtctttttcgtgacattcagttcgctgcgctcacggctctggcagtgaatgggggtaaatggcactacaggcgccttttatggattcatgcaaggaaactacccataatacaagaaaagcccgtcacgggcttctcagggcgttttatggcgggtctgctatgtggtgctatctgactttttgctgttcagcagttcctgccctctgattttccagtctgaccacttcggattatcccgtgacaggtcattcagactggctaatgcacccagtaaggcagcggtatcatcaacaggcttacccgtcttactgtccctagtgcttggattctcaccaataaaaaacgcccggcggcaaccgagcgttctgaacaaatccagatggagttctgaggtcattactggatctatcaacaggagtccaagcgagctcgatatcaaattacgccccgccctgccactcatcgcagtactgttgtaattcattaagcattctgccgacatggaagccatcacaaacggcatgatgaacctgaatcgccagcggcatcagcaccttgtcgccttgcgtataatatttgcccatggtgaaaacgggggcgaagaagttgtccatattggccacgtttaaatcaaaactggtgaaactcacccagggattggctgagacgaaaaacatattctcaataaaccctttagggaaataggccaggttttcaccgtaacacgccacatcttgcgaatatatgtgtagaaactgccggaaatcgtcgtggtattcactccagagcgatgaaaacgtttcagtttgctcatggaaaacggtgtaacaagggtgaacactatcccatatcaccagctcaccgtctttcattgccatacgaaattccggatgagcattcatcaggcgggcaagaatgtgaataaaggccggataaaacttgtgcttatttttctttacggtctttaaaaaggccgtaatatccagctgaacggtctggttataggtacattgagcaactgactgaaatgcctcaaaatgttctttacgatgccattgggatatatcaacggtggtatatccagtgatttttttctccattttagcttccttagctcctgaaaatctcgataactcaaaaaatacgcccggtagtgatcttatttcattatggtgaaagttggaacctcttacgtgccgatcaacgtctcattttcgccagatatcgacgtcggtgcctaatgagtgagctaacttacattaattgcgttgcgctcactgcccgctttccagtcgggaaacctgtcgtgccagctgcattaatgaatcggccaacgcgcggggagaggcggtttgcgtattgggcgccagggtggtttttcttttcaccagtgagacgggcaacagctgattgcccttcaccgcctggccctgagagagttgcagcaagcggtccacgctggtttgccccagcaggcgaaaatcctgtttgatggtggttaacggcgggatataacatgagctgtcttcggtatcgtcgtatcccactaccgagatgtccgcaccaacgcgcagcccggactcggtaatggcgcgcattgcgcccagcgccatctgatcgttggcaaccagcatcgcagtgggaacgatgccctcattcagcatttgcatggtttgttgaaaaccggacatggcactccagtcgccttcccgttccgctatcggctgaatttgattgcgagtgagatatttatgccagccagccagacgcagacgcgccgagacagaacttaatgggcccgctaacagcgcgatttgctggtgacccaatgcgaccagatgctccacgcccagtcgcgtaccgtcttcatgggagaaaataatactgttgatgggtgtctggtcagagacatcaagaaataacgccggaacattagtgcaggcagcttccacagcaatggcatcctggtcatccagcggatagttaatgatcagcccactgacgcgttgcgcgagaagattgtgcaccgccgctttacaggcttcgacgccgcttcgttctaccatcgacaccaccacgctggcacccagttgatcggcgcgagatttaatcgccgcgacaatttgcgacggcgcgtgcagggccagactggaggtggcaacgccaatcagcaacgactgtttgcccgccagttgttgtgccacgcggttgggaatgtaattcagctccgccatcgccgcttccactttttcccgcgttttcgcagaaacgtggctggcctggttcaccacgcgggaaacggtctgataagagacaccggcatactctgcgacatcgtataacgttactggtttcacattcaccaccctgaattgactctcttccgggcgctatcatgccataccgcgaaaggttttgcgccattcgatggtgtccgggatctcgacgctctcccttatgcgactcctgcattaggaagcagcccagtagtaggttgaggccgttgagcaccgccgccgcaaggaatggtgcatgcaaggagatggcgcccaacagtcccccggccacggggcctgccaccatacccacgccgaaacaagcgctcatgagcccgaagtggcgagcccgatcttccccatcggtgatgtcggcgatataggcgccagcaaccgcacctgtggcgccggtgatgccggccacgatgcgtccggcgtagaggatcgagaattgtgagcggataacaattgacattgtgagcggataacaagatactgagcacatcagcaggacgcactgaccgaattcaaaagatctgatacctctccctgggaagagaatatggtgagcaagggcgaggagctgttcaccggggtggtgcccatcctggtcgagctggacggcgacgtaaacggccacaagttcagcgtgtccggcgagggcgagggcgatgccacctacggcaagctgaccctgaagttcatctgcaccaccggcaagctgcccgtgccctggcccaccctcgtgaccaccctgacctacggcgtgcagtgcttcagccgctaccccgaccacatgaagcagcacgacttcttcaagtccgccatgcccgaaggctacgtccaggagcgcaccatcttcttcaaggacgacggcaactacaagacccgcgccgaggtgaagttcgagggcgacaccctggtgaaccgcatcgagctgaagggcatcgacttcaaggaggacggcaacatcctggggcacaagctggagtacaactacaacagccacaacgtctatatcatggccgacaagcagaagaacggcatcaaggtgaacttcaagatccgccacaacatcgaggacggcagcgtgcagctcgccgaccactaccagcagaacacccccatcggcgacggccccgtgctgctgcccgacaaccactacctgagcacccagtccgccctgagcaaagaccccaacgagaagcgcgatcacatggtcctgctggagttcgtgaccgccgccgggatcactctcggcatggacgagctgtacaagtaa

**References**

1. Bálint Csörgő, Tamás Fehér, Edit Tímár, Frederick R Blattner, Pósfai G. 2012. Low-mutation-rate, reduced-genome Escherichia coli: an improved host for faithful maintenance of engineered genetic constructs. Microbial Cell Factories 11.

2. Hartline CJ, Zhang R, Zhang F. 2022. Transient Antibiotic Tolerance Triggered by Nutrient Shifts From Gluconeogenic Carbon Sources to Fatty Acid. Front Microbiol 13:854272.
